# Multifocal Organoid Capturing of Colon Cancer Reveals Pervasive Intratumoral Heterogenous Drug Responses

**DOI:** 10.1002/advs.202103360

**Published:** 2021-12-17

**Authors:** Soon‐Chan Kim, Ji Won Park, Ha‐Young Seo, Minjung Kim, Jae‐Hyeon Park, Ga‐Hye Kim, Ja Oh Lee, Young‐Kyoung Shin, Jeong Mo Bae, Bon‐Kyoung Koo, Seung‐Yong Jeong, Ja‐Lok Ku

**Affiliations:** ^1^ Korean Cell Line Bank Laboratory of Cell Biology Cancer Research Institute Seoul National University College of Medicine Seoul 03080 South Korea; ^2^ Department of Biomedical Sciences Seoul National University College of Medicine Seoul 03080 South Korea; ^3^ Cancer Research Institute Seoul National University Seoul 03080 South Korea; ^4^ Ischemic/Hypoxic Disease Institute Seoul National University College of Medicine Seoul 03080 South Korea; ^5^ Department of Surgery Seoul National University College of Medicine Seoul 03080 South Korea; ^6^ Division of Colorectal Surgery Department of Surgery Seoul National University Hospital Seoul 03080 South Korea; ^7^ Department of Pathology Seoul National University College of Medicine Seoul 03080 South Korea; ^8^ Institute of Molecular Biotechnology of the Austrian Academy of Sciences (IMBA) Vienna Biocenter (VBC) Dr. Bohr‐Gasse 3 Vienna 1030 Austria

**Keywords:** colorectal cancer, drug responses, intratumor heterogeneity, patient‐derived organoid

## Abstract

Intratumor heterogeneity (ITH) stands as one of the main difficulties in the treatment of colorectal cancer (CRC) as it causes the development of resistant clones and leads to heterogeneous drug responses. Here, 12 sets of patient‐derived organoids (PDOs) and cell lines (PDCs) isolated from multiple regions of single tumors from 12 patients, capturing ITH by multiregion sampling of individual tumors, are presented. Whole‐exome sequencing and RNA sequencing of the 12 sets are performed. The PDOs and PDCs of the 12 sets are also analyzed with a clinically relevant 24‐compound library to assess their drug responses. The results reveal unexpectedly widespread subregional heterogeneity among PDOs and PDCs isolated from a single tumor, which is manifested by genetic and transcriptional heterogeneity and strong variance in drug responses, while each PDO still recapitulates the major histologic, genomic, and transcriptomic characteristics of the primary tumor. The data suggest an imminent drawback of single biopsy‐originated PDO‐based clinical diagnosis in evaluating CRC patient responses. Instead, the results indicate the importance of targeting common somatic driver mutations positioned in the trunk of all tumor subregional clones in parallel with a comprehensive understanding of the molecular ITH of each tumor.

## Graphical abstract

There is an imminent drawback of patient‐derived organoid‐based clinical diagnosis from single biopsy in evaluating colorectal cancer patient responses. The data suggest the importance of targeting ancestral somatic driver mutations positioned in the trunk of all tumor subregional clones in parallel with a comprehensive understanding of the molecular intratumor heterogeneity of each tumor in order to defy potential drug resistance.

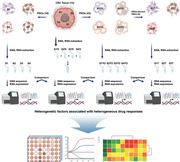

## 1 Introduction

Colorectal cancer (CRC) is the third most commonly diagnosed human malignancy worldwide and represents the third most common cause of tumor‐associated mortalities in Korea.^[^ ^1^ ^]^ Regardless of the clinical accomplishments of early detection and prevention that have brought about a general decrease of CRC incidence,^[^ ^2^ ^]^ acquired resistance to chemo‐ and targeted therapeutics remains the main cause of advanced CRC‐associated morbidity, with scarcely any remedial options available.^[^ ^3^ ^]^ Intratumor heterogeneity (ITH) and cancer clonal evolution have received increased attention since ITH acquired throughout cancer progression seemingly contributes to therapeutic resistance and therefore a dismal oncologic outcome.^[^ ^4^ ^]^ Genetic intratumor heterogeneity has been reported in several types of solid tumors, such as renal,^[^ ^5^ ^]^ breast,^[^ ^6^ ^]^ esophageal,^[^ ^7^ ^]^ lung,^[^ ^8^ ^]^ ovarian,^[^ ^9^ ^]^ prostate,^[^ ^10^ ^]^ and pancreatic^[^ ^11^ ^]^ tumors. Multiregional sequencing of spatiotemporally distinct tumor regions makes it possible to follow the evolutionary trajectories of cancer cells, with common ancestral somatic driver mutations positioned within the whole tumor mass, but also with subregional mutations that are restricted to one of the subregional clones. The parental clone develops all the common mutations inherited to subregional clones and the accumulation of subregional genetic changes eventually develops into ITH, which is thought to be the main cause of poor therapeutic responses.^[^ ^12^ ^]^

CRC presents a high spatial heterogeneity but its implication in heterogeneous drug responses has not been fully investigated. Although multiregional analysis of CRC has been conducted in many groups,^[^ ^13^ ^]^ sequencing approaches using primary cancer tissues have substantial limitations in connecting molecular ITH to various drug responses. In this respect, the unique ability of organoid technology to capture ITH by establishing independent subregional clones from multiple regions of a single tumor mass is valuable for simultaneously assessing both genetic heterogeneity and its impact on diverse drug responses.^[^ ^14^ ^]^ Nevertheless, previous CRC studies using organoid technology have mainly focused on the comparison between drug responses of primary and metastatic tumor‐derived organoids.^[^ ^15^ ^]^ A few studies have associated the significance of ITH with drug responses of CRC and showed its implications for precision medicine,^[^ ^16^ ^]^ yet the number of patients analyzed by subclonal or subregional organoids derived from a single tumor mass, as well as the number of drugs in the screening panel, were insufficient to draw generalizable conclusions. Besides, the heterogeneous drug responses within a single patient in perspective of genetic and transcriptomic ITH has been seldom interpreted. To obtain a clearer overview of the impact of ITH on heterogeneous drug responses, we prepared 12 sets of PDOs and PDCs consisting of 3–4 subregional clones isolated from multiple regions of single tumors from 12 patients, which were then subjected to massive genomic, transcriptomic, and pharmacological analyses.

Our result not only showed an unexpectedly pervasive high level of heterogeneity of drug responses caused by various molecular heterogeneities among subregional clones in all 12 patients but also provided clone‐by‐clone comparisons in perspective of molecular ITH to clarify heterogeneous drug responses, and enabled comprehensive prediction of subregional drug resistance by integrating genetic and transcriptomic heterogeneity. Our study also demonstrates the limitations of single biopsy‐based precision medicine screening and highlights the importance of targeting trunk mutations shared by all cancer subregional clones in parallel with comprehensive integration of transcriptomic and proteomic heterogeneity with genomic factors in order to prevent unwanted drug resistance caused by ITH.

## 2 Results

### 2.1 Subregional Colorectal Cancer Organoids and Cell Lines from 12 Patients Retained the Histological Features of the Original Tumor

**Figure 1:**
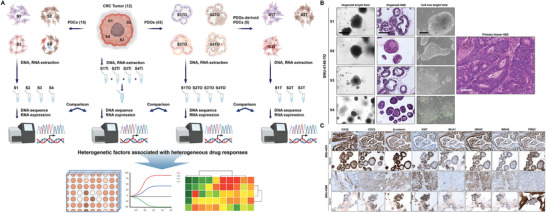
Histopathological characterization of patient‐derived organoids and cell lines. See also Figures S1 and S2 in the Supporting Information. A) Overall study design. We successfully generated 43 PDOs and 23 PDCs (15 PDCs and 8 PDO culture‐derived PDC cultures) corresponding to 12 different patients. We compared multiomics data from all subregional derivatives, and analyzed heterogenetic factors that might affect drug responses. B) Organoid architecture resembles primary tumor epithelium. Most organoids had one or more lumens, resembling the tubular structures of the primary tumor. Tumors devoid of a lumen give rise to compact organoids without the lumen. Hematoxylin‐eosin (H&E) staining of formalin‐fixed paraffin‐embedded (FFPE) organoid sections revealed that tumor‐derived organoids presented patient‐specific heterogeneous morphologies. Organoid (*n* = 43) bright‐field and H&E scale bar = 250 × 10^−6^ m. Tissue (*n* = 12) scale bar = 105 × 10^−6^ m. Cell line (*n* = 21) bright‐field scale bar = 70 × 10^−6^ m. C) Cytokeratin 20 (*CK20*) and caudal type homeobox 2 (*CDX2*), as well as nuclear *β*‐catenin (*CTNNB1*) and KI‐67 (*MKI67*), were compared between PDOs and parental tumors using immunohistochemistry. Also, the expressional patterns of MMR genes such as MutL homolog 1 (*MLH1*), MutS Homolog 2 (*MSH2*), MutS Homolog 6 (*MSH6*), and PMS1 Homolog 2 (*PMS2*) were compared between PDOs and matched tumors. PDOs retained similar levels of these markers as the parental tumors. Scale bar = 200 × 10^−6^ m.

We have established a living biobank of patient‐derived colorectal cancer organoids (PDOs) and cell lines (PDCs), which will be deposited at the Korean Cell Line Bank (http://cellbank.snu.ac.kr) to be distributed worldwide. The multifocal regions of the primary tumor were designated as S1–S4 before preprocessing for further culture. We successfully generated 43 subregional PDOs and 23 subregional PDCs (eight of which were derived from PDOs rather than directly) corresponding to 12 different patients. This allowed us to biologically capture the ITH of each cancer by multifocal sampling. Subsequently, we performed genomic and transcriptomic analyses of the set from each patient, as well as drug responses to a panel of 24 selected drugs, in order to understand the impact of molecular ITH on the biological responses to cancer therapeutics (overall study design in **Figure** **1**A).

In this study, we used suboptimal culture medium that completely prevents the propagation of normal colon cells within three passages (more than 100 samples tested) (Figure S1A,B, Supporting Information). **Table** **1** summarizes the clinicopathological information of all 12 patients enrolled in this study. Fingerprinting analysis indicated that in all cases the multifocal PDOs and PDCs derived from the same patient shared >90% of specific loci and were not cross‐contaminated (Table S1A, Supporting Information). We also applied our RNA‐sequencing data to authenticate that our derivatives were not mixed before sequencing (Table S1B, Supporting Information). The variant profiles of each patient were distinct and overall shared low variant identity with other patients whereas multiregional lines exhibited significantly high correlation score, which suggested that there was no mixing of the lines before sequencing. In line with previous data,^[^ ^14^ ^]^ PDOs varied in their growth rate and morphology (Figure 1B; Figure S1C–O, Supporting Information), e.g., spheroidal, asymmetric, or loosely aggregated. The matched PDCs grew as monolayers of substrate‐adherent cells displaying polygonal and spindle morphology (Figure 1B; Figure S1E–O, Supporting Information). Hematoxylin‐eosin (H&E) staining of paraffin sections from the PDOs, as well as the corresponding tumors, exhibited a strong concordance in their histopathological features (Figure 1B; Figure S1E–O, Supporting Information). Cytokeratin 20 (*CK20*) and caudal type homeobox 2 (*CDX2*), as well as nuclear *β*‐catenin (*CTNNB1*) and KI‐67 (*MKI67*), were compared between PDOs and corresponding tumors. Also, the expression patterns of mismatch repair (MMR) genes such as MutL homolog 1 (*MLH1*), MutS Homolog 2 (*MSH2*), MutS Homolog 6 (*MSH6*), and PMS1 Homolog 2 (*PMS2*) were compared between PDOs and matched tumors. PDOs retained analogous intensity and expression of these markers (Figure 1C; Figure S2A–J, Supporting Information). Two MSI‐H tumors (SNU‐4376A and SNU‐4398) were deficient in *MLH1* and *PMS2* expression (Figure 1C; Figure S2D, Supporting Information). Overall, these data validated that the histological features and expression patterns of specific CRC markers are well conserved in the PDOs. We further inspected biological signs of ITH among each set of established PDOs. Even though derived from the same tumor mass, SNU‐4849S1‐TO, one of the subregional clones derived from patient SNU‐4849, exhibited a much slower growth rate compared to the other subregional clones from the same origin. There was also intramorphological heterogeneity in the subregional clone, SNU‐4374S4‐TO. One subregional clone had a spheroidal and asymmetric shape, whereas another subregional clone retained a thin‐walled cystic structure with a lumen, as confirmed by H&E staining as well as immunofluorescence imaging (Figure S1D,G, Supporting Information). Taken together, these data validated that we have successfully established 12 sets of subregional PDO as well as PDC lines that retain the original characteristics of the isolated tumors with signs of biological variances among cancer subregional clones from the same origin.

**Table 1:** Clinicopathological information of 12 CRC samples

SNU number	Sex/age	MSI^a)^	p T	p N	p M	PreTx CEA^b)^	Tumor size [cm]	Stage	Tumor Loc.	Met Loc.^c)^	Rec Loc.^d)^	Regimen
SNU‐4139	F/38	MSI‐L	4	1	1	16.7	8	4	Sigmoid colon	Liver, lung, bone, p‐seeding		N/a
SNU‐4146	M/81	MSI‐L	3	1	0	0.9	5.5	3	Sigmoid colon			Xeloda^e)^
SNU‐4351	F/60	MSS	3	0	0	8.4	3.5	2	Sigmoid colon			5‐FU(LV)^f)^
SNU‐4374	F/66	MSS	3	0	0	8.5	3.7	2	Descending colon			5‐FU(LV)^f)^
SNU‐4376A	M/39	MSI‐H	4	0	1	12.2	12	4	Transverse colon	Liver		Xelox^g)^
SNU‐4398	F/83	MSI‐H	3	0	0	3.3	3.1	2	Ascending colon			N/a
SNU‐4631A	F/75	MSS	4	2	0	1.5	8.5	3	Ascending colon			Xelox^g)^
SNU‐4646	M/86	MSS	3	1	0	3.3	7	3	Rectum		Lung	N/a
SNU‐4713	F/86	MSS	3	0	0	3.7	5	2	Sigmoid colon			N/a
SNU‐4796	M/70	MSS	3	0	0	1.3	4.5	2	Ascending colon			Xeloda^e)^
SNU‐4813	M/77	MSS	3	0	0	2.3	7.5	2	Sigmoid colon			N/a
SNU‐4849	M/82	MSS	3	0	0	2.7	4	2	Ascending colon			N/a

^a)^ MSI indicates microsatellite instability

^b)^ PreTx CEA indicates pretreatment carcinoembryonic antigen

^c)^ Met Loc. indicates metastasis location

^d)^ Rec Loc. indicates recurrence location

^e)^ Xeloda indicates capecitabine

^f)^ 5‐FU(LV) indicates 5‐fluorouracil/leucovorin

^g)^ Xelox indicates oxaliplatin/capecitabine.

### 2.2 Subregional Organoids and Cell Lines Recapitulate the Genomic Features with Moderate Intratumor Heterogeneity of Human Colorectal Cancer

**Figure 2:**
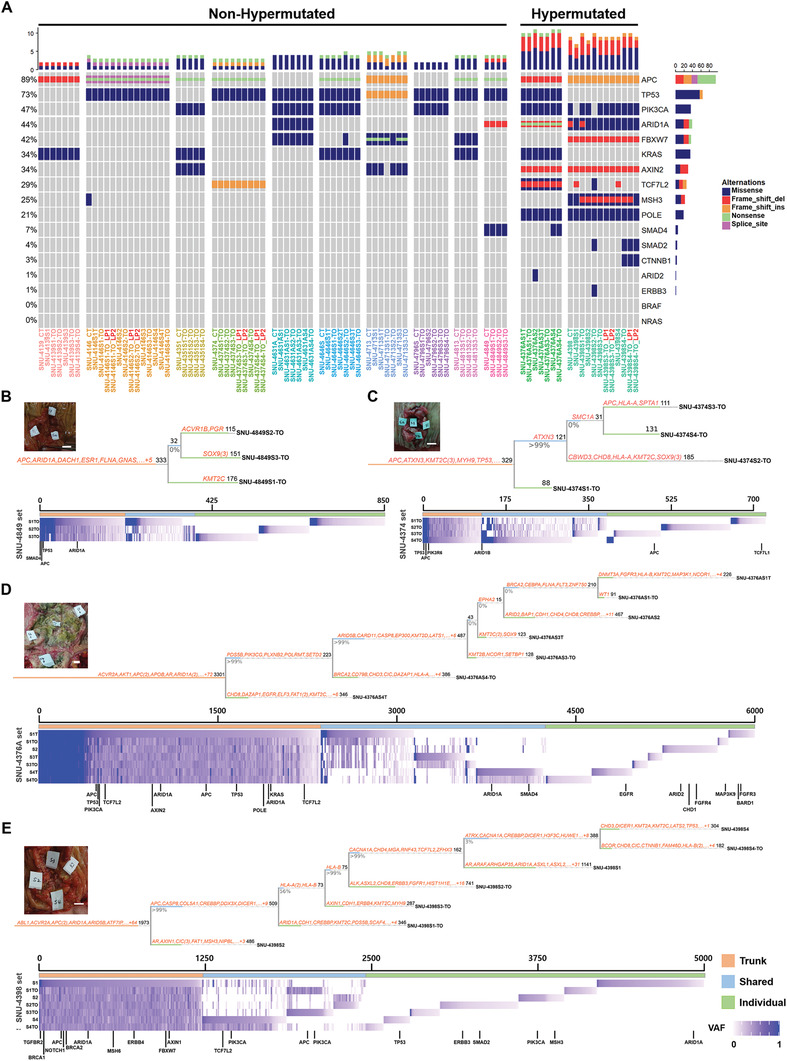
Subregional analysis of the genomic features of patient‐derived organoids and cell lines reveals evolutionary trajectories of 12 CRCs. See also Figures S3–S5 in the Supporting Information. A) Heatmap of mutational variations among the frequently mutated genes in CRC. WES identified multiple somatic mutations including point mutations in putative tumor driver genes in the 12 CRC series, which together consisted of 42 PDOs, 23 PDCs, and 11 tumor samples. The recurrently mutated genes in CRC were recapitulated in the PDOs and PDCs. Derivatives from patient SNU‐4376A and SNU‐4398 exhibited features of hypermutation (>10 mutations Mb^−1^). Derivatives are marked with representative colors in accordance with their patient origin. The derivatives ending with _LP indicate subregional clones at initial passage. B,C) Phylogenetic trees demonstrating the evolutionary history of two MSS CRC sets. D,E) Phylogenetic trees illustrating the evolutionary trajectory of two MSI‐H CRC sets. Based on the multiregional WES profiles, Treeomics classified somatic mutations as trunk (orange), shared (blue), and individual (green). Numbers on top of each branch indicate the number of acquired variants. Genes that are listed in the CGC are shown in orange. Numbers on the bottom of each branch indicate the estimated support values. The actual photograph of each tumor shows the marked sites from which each subregional derivative was obtained (S1–S4). Scale bars = 1 cm. Multiregional variant allele frequencies (VAFs) are shown as a heatmap under the phylogenetic tree. The three different classes of mutations (trunk, shared, and individual) are indicated by the colored bars on the top. Driver mutations are indicated below each heatmap. The Treeomics statistical settings were identical for all samples (sequencing error rate = 0.005, prior absent probability = 0.5, max absent VAF = 0.05, LOH frequency = 0, false discovery rate = 0.05, false‐positive rate = 0.005, and absent classification minimum coverage = 100).

Several studies have shown that PDOs and PDCs retain the genomic landscape of the original tumor, including somatic mutations and copy number variations (CNVs).^[^ ^15^, ^17^ ^]^ We performed whole‐exome sequencing (WES) on multifocal samples derived from 12 different patients to determine the genomic concordance between parental tumors and its derivatives. For each patient, we sequenced three to four multiregional PDOs, PDCs, a matched tumor tissue and normal mucosa as a control. Although normal mucosa has been shown to contain some cancer‐relevant somatic aberrations and transcriptomic features,^[^ ^18^ ^]^ our study mainly focused on the comparison between subregional clones using variant allele frequencies (VAFs) of driver mutations, and therefore the minor contamination in those normal controls was considered negligible. Cancer cell evolution may occur as a result of positive clonal selection that is partially sensitive to culture conditions.^[^ ^19^ ^]^ In order to assure that the observed ITH among different subregional clones was not caused by culture‐associated genetic mutations, we performed additional WES of a few subregional clones in their low passages (p1‐2, ending with _LP). We compared driver mutations, mutational signatures, CNVs and drug responses of those early passage subregional sets, including two microsatellite stable (MSS) tumors (two patients) and one MSI tumor (one patient). Our results indicate that the observed genetic heterogeneity among subregional clones was well conserved between early and late passages (**Figure** **2**A; Figures S3 and S4, Supporting Information), suggesting that most of the observed ITH is of tumor origin.

WES identified multiple mutations in the samples, including point mutations in putative oncogenes. The mutational profiles of the samples are summarized in Table S2A,B in the Supporting Information. Two patients (SNU‐4376A and SNU‐4398) showed features of hypermutation (>10 mutations Mb^−1^) (Figure 2A). Frequent mutations commonly observed in CRC^[^ ^20^ ^]^ were well represented in our PDO and PDC sets. These include inactivating mutations in tumor suppressors such as *TP53*, *APC*, and *FBXW7*, as well as activating alterations in *KRAS* (codon 12) and *PIK3CA* (codon 107, 542, 545, 939, 1044, and 1047) (Figure 2A; Table S2A,B, Supporting Information). Genetic alterations in MMR‐associated pathways are concomitant with the hypermutated phenotype.^[^ ^21^ ^]^ The derivatives of two microsatellite instability‐high (MSI‐H) patients (SNU‐4376A and SNU‐4398) harbored missense mutations in the *POLE* gene, with SNU‐4398 having an additional point mutation in *MSH3*, in concordance with their classification as hypermutated CRC.^[^ ^22^ ^]^ The majority of CRC cases harbored activating mutations in *CTNNB1* or inactivating mutations in *APC*, *AXIN2*, *FBXW7*, or *FAM123B*.^[^ ^22^ ^]^ Among our non‐MSI‐H types, we found *APC* alterations in all but two patients (SNU‐4631A and SNU‐4796). None carried activating mutations in *CTNNB1*. The SNU‐4631A had a missense mutation in *FBXW7*. Epigenetic regulation takes a significant role in the initiation and progression of CRC.^[^ ^22^ ^]^ In our cohort, various genes related to the epigenome were mutated at a high rate, such as *ARID1A*, *KMT2C*, *KMT2D*, and *KDM6A*, which are commonly altered in CRC.^[^ ^23^ ^]^ Overall, the mutational spectrums identified in our PDOs and PDCs reflected the genomic features of their parental tumors (Figure 2A; Table S2A,B, Supporting Information).

The mutational concordance of the subregional sets within the coding regions corresponded closely with the matched tumor specimens for both the hypermutated and nonhypermutated patients as expected (median = 0.89 frequency of concordance ranging 0.77 to 0.94 (Figure S3A and Table S3A, Supporting Information)). We analyzed discordant alterations between derivatives and matched tissues to assess the level of ITH in our subregional sets compared to their parent tumor. On average, 5.3% (43/798) of discordant mutations were exclusively present in PDOs and PDCs (Table S3B, Supporting Information). These are expected to be fractional mutations enriched in the subregional lines, which were present at low frequencies in the original tumor. Importantly, the concordance of mutational features in the low passage organoids excludes the possibility of de novo gain of these mutations during culture establishment and subsequent passaging (Figure S3A, Supporting Information). On the other hand, 3.4% (29/844) of discordant mutations were detected solely in tumor tissue (Table S3C, Supporting Information). These tumor‐only discordant mutations had a relatively low allelic frequency in the tumor tissue, suggesting the possibility that our multiregion sampling method still misses some subregional mutations due to the limited sample number (*n* = 3–4). We then looked at the mutational signatures of the subregional sets in comparison to the parent tumors. The most predominant point mutation type in total samples was the C‐to‐T transition (Table S3D, Supporting Information), closely matching the other large cohort CRC sequencing results.^[^ ^24^ ^]^ The mutational signature patterns of PDOs and PDCs originating from the same patient were concordant with moderate subregional variations (Figure S3B and Table S3D, Supporting Information).

We also compared the exome‐wide CNVs of PDOs and PDCs to the parent tumor tissues, except for the SNU‐4351 and SNU‐4796 sets, in which the primary tumor specimens and matched normal samples were of insufficient purity to determine the CNVs. Subregional PDOs and PDCs displayed mostly analogous CNV patterns to the parental tumors (Figure S4A–J, Supporting Information). Several CNVs were only observed on selected chromosomes of some subregional clones, such as gain at chromosome 7 and loss at chromosome 14 of the SNU‐4146S1‐TO sample. We again confirmed that those chromosomal alternations were already present at the initial passages (Figure S4B,C,E, Supporting Information). Our samples displayed comparable CNVs with a much larger clinical cohort^[^ ^24^ ^]^ Inspection of the top regions identified by TCGA revealed the presence of MYC‐ and BRCA2‐amplified and SMAD4‐depleted PDOs and PDCs, as well as a documented gain of the 13q region in the nonhypermutated group (Figure S4L, Supporting Information). Overall, this data confirms that the subregional PDOs and PDCs recapitulate the genomic characteristics of the primary tumor and most of the genomic diversity of CRC. It also shows that our multiregional sampling is a valid method for capturing the genomic ITH of the original tumor, as suggested in previous reports.^[^ ^16a,b^ ^]^

### 2.3 Tracking Evolutionary Histories of 12 Multisampling CRC Sets Revealed Significant Genomic Heterogeneity

We applied the Treeomics algorithm^[^ ^25^ ^]^ to the multiregional sequencing data to draw evolutionary trajectories. We included PDOs as well as PDCs to determine if the different culture methods affect the mutational aspects. We rectified any possible sequencing artifacts with the Treeomics algorithm to make the mutational patterns of each sample compatible with the topological variant of the evolutionary tree. Based on multiregional WES profiles, Treeomics classified somatic mutations as all (trunk), more than two samples (shared) and a single sample (individual) (Figure 2B–E; Figure S5A–H, Supporting Information). Our cohort involved organoid‐derived cell lines (ending with –T), which were suitable for detecting culture‐associated mutations. Common driver genes with potential functional mutations, including nonsynonymous single‐nucleotide variants, stop‐gain SNVs, splicing SNVs, or insertion/deletions (indels) were plotted for analyzing the evolutionary history of each tumor. For instance, SNU‐4849 harbored multiple tumor driver mutations, such as *APC* (c.4132C > T/p.Q1378*), *TP53* (c.713G > A/p.C238Y), and *ARID1A* (c.4187_4188del/p.G1396Afs*48), with an allele frequency of ≈0.95 in the trunk, while each of the subregional clones shaped the phylogenetic tree with individual mutations of ≈0.2 VAFs (Figure 2B; Table S2B, Supporting Information). This implies that the first hit mainly contributed to the tumorigenesis of epithelial cells, and thereafter the tumor had not progressed. The SNU‐4374 tree had a similar shape to SNU‐4849, and displayed several driver mutations as well. The SNU‐4374 series had several driver mutations as well, such as *APC* (c.4132C > T/p.Q1378*), *TP53* (c.406C > G/p.Q136E) with VAFs of ≈0.95 in the trunk while each of the subregional clones outlined the shape of the tree with individual mutations with VAFs of ≈0.3. Nevertheless, the SNU‐4374S2‐TO clone had a protruding acquisition of a mutational burden in the *KMT2C* and *SOX9* genes with VAFs of ≈0.35, which suggests one major branch was formed by epigenetic factors (Figure 2C; Table S2B, Supporting Information).

The original tumor tissue of SNU‐4146 had a *TP53* mutation (c.817C > T/p.R273C) with VAF of 1, which appeared in a relatively early stage of the tumor evolution, and two *APC* mutations (c.637C > T/p.R213* and c.1312+2T > G/p.X438_splice) with VAFs of 0.51 and 0.43, respectively (Figure S5B, Supporting Information). We observed that VAFs of nonsense *APC* mutations (c.637C > T/p.R213*) were increased to 0.71 and 0.82 in subregional clones SNU‐4146S2‐TO and SNU‐4146S3‐TO, respectively, whereas VAFs of the other two subregional clones remained unchanged. In contrast, the splice site *APC* mutation (c.1312+2T > G/p.X438_splice) had decreased VAFs of 0.37 and 0.26 in subregional clones SNU‐4146S2‐TO and SNU‐4146S3‐TO, respectively (Figure S5B and Table S2B, Supporting Information). VAFs of the other two subregional clones remained unchanged as well. This analysis revealed that, even though two *APC* mutations were present in all subregional clones, two major subregional clones were subject to a loss of heterozygosity (LOH), leading to biallelic inactivation of *APC*. We also made a direct comparison of molecular features between cell lines when established directly from the patient (SNU‐4146S4) and adapted from the organoid (SNU‐4146S4T). We expected the organoid‐derived cell line likely have higher VAFs in driver mutations since they underwent more selective forces than the tissue‐derived cell line, yet the VAFs of driver mutations were nearly identical in those cell lines (Table S2B, Supporting Information). The overall mutational concordance of SNU‐4146S4 and SNU‐4146S4T were also comparable, which was 0.79 and 0.78, respectively (Table S3A, Supporting Information). This suggested that the mutational predisposition of these two cell lines was likely determined by the subregional heterogeneity rather than the different culture methods. We further validated the specific subregional distance between two cell lines by constructing the phylogenetic tree of SNU‐4146 patient including both cell lines (Figure S3C, Supporting Information). This analysis revealed that SNU‐4146S4 and SNU‐4146S4T were adjacent to each other on the phylogenetic tree, which implies they share the majority of subregional mutations. Especially, both cell lines harbored *CBWD3* mutation (c.394G > T/p.D132Y) which was absent in SNU‐4146S4‐TO.

Nearly 50% of the mutations of two MSI‐H tumor derivative series (SNU‐4376A and SNU‐4398 sets) occurred in the same genes. VAFs of major hit mutations including *APC*, *TP53*, *PIK3CA*, and *KRAS* were relatively low in the MSI‐H series (Figure 2D,E). There were discordant driving alterations in *APC* and *TP53*, suggesting that hypermutated phenotypes may have been present prior to the LOH, leading to the biallelic inactivation of *APC* and *TP53* and the acquisition of growth‐promoting mutations. Another 10 MSS tumor‐derived sets share at least one major hit with VAFs of ≈0.95, which indicates that the somatically mutated progenitor cells had been mutually ancestral but then diverged to acquire independent secondary alterations (Figure 2B,C; Figure S5 and Table S2B, Supporting Information).

Multifocal capturing of CRC makes it possible to detect a series of genes that account for not only the initiation but also the evolution of colon tumors. Two MSI patients were not included due to the high tumor mutational burden. To exclude random somatic mutations, only genes found to be mutated in more than three patients were counted. A total of 78 trunk genes that are potentially associated with tumor initiation (Figure S6A and Table S4A, Supporting Information) and 444 shared/individual genes that shaped tumor evolution were mutated (Figure S6B and Table S4B, Supporting Information). Genes that are cross‐listed in the Cancer Gene Census (CGC)^[^ ^26^ ^]^ are highlighted in red. Genes present in the Vogelgram,^[^ ^27^ ^]^ such as *APC*, *TP53*, *KRAS*, and *FBXW7* were exclusively found among the 78 tumor‐initiating genes, indicating that in our samples Vogelgram mutations mainly played a role in tumor initiation and had less influence on subregional evolution. Only six genes were present in both tumor initiation and tumor evolution stage: *MUC4*, *MUC16*, *USP6*, *BCLAF1*, *KMT2C*, and *NOTCH2*. Those genes might account for the mutational trajectory of tumor subregional clones.

### 2.4 Gene Expression Analysis Reveals Substantial Transcriptomic Heterogeneity in 12 Multisampling CRC Tumors

**Figure 3:**
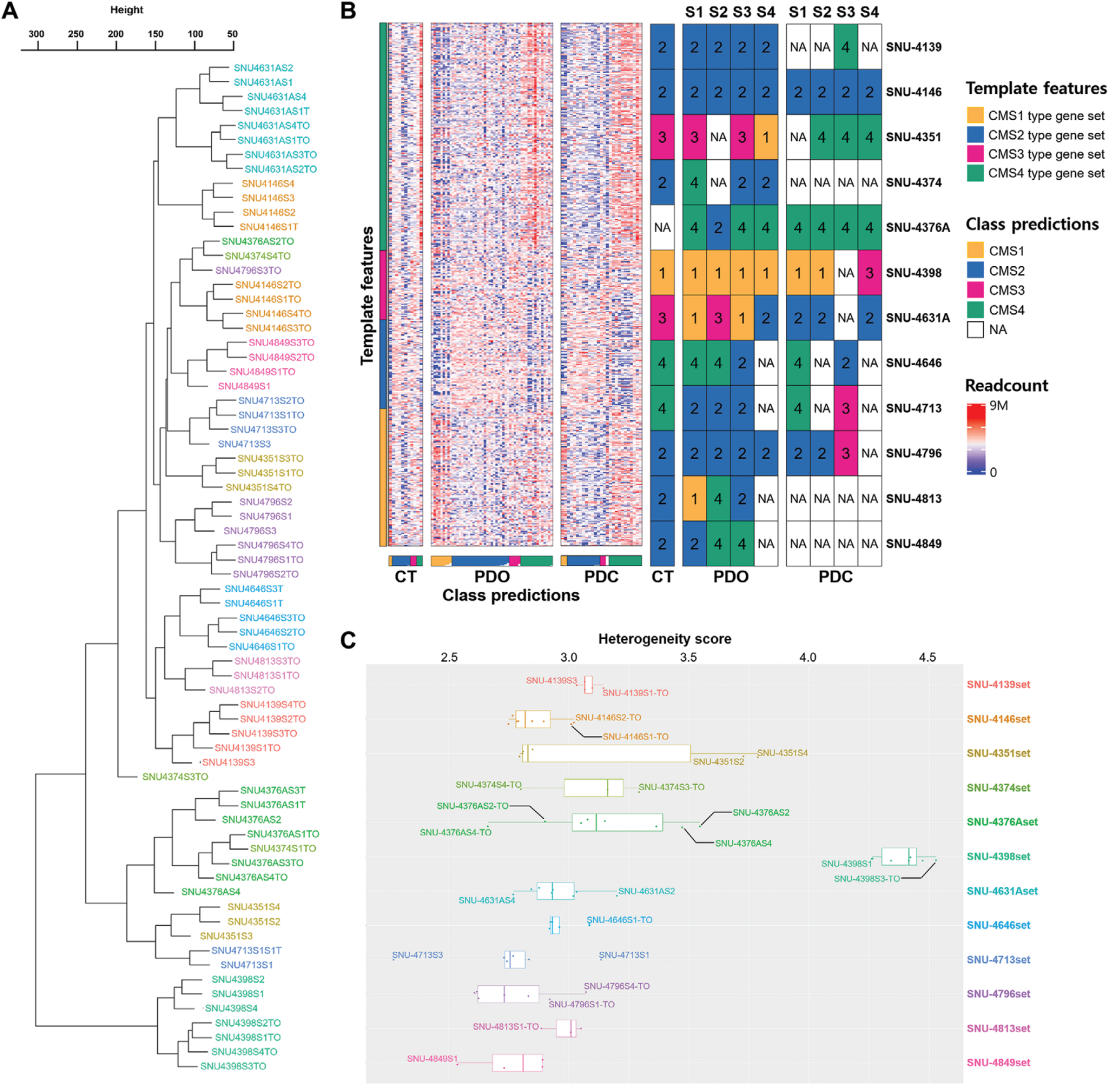
Transcriptome analysis validated significant heterogeneity in 12 multisampling CRC tumors. A) Hierarchical clustering analysis of subregional derivatives (*n* = 70). Euclidean distance (height) of mRNA expressions indicated that the effect of different culture methods as well as interpatient heterogeneity on mRNA expression profiles of each subregional clone was substantial with few exceptions. Derivatives are marked with representative colors in accordance with the patient origin. Unsupervised Ward's minimum variance method was used as the distance metric for clustering. B) CMS subtyping of subregional derivatives (*n* = 67) and original tissues (*n* = 11). Heterogeneous distribution of the CMS types was observed in most of multifocal sampling CRC sets. The original CMS type of the tumor was better retained in PDOs than PDCs. Subtype‐specific gene sets with accordant class predictions are marked with symbolic colors for each CMS subtype (yellow: CMS1; blue: CMS2; red: CMS3; green: CMS4). The multifocal subregional clone numbers (S1–S4) are indicated on the top. Unavailable subregional samples are marked with NA. C) Box plot of mRNA heterogeneity scores (DEPTH) showing that SNU‐4374, SNU‐4376A, and SNU‐4796 sets had high transcriptional ITH. Subregional PDOs (*n* = 43) and PDCs (*n* = 28) derived from the same patient are marked with representative colors.

We verified that the patterns of mRNA expression reflect the ITH as well. We validated that there was no mixing of lines before sequencing (Table S1B, Supporting Information), which excluded the potential misinterpretation of mixed sequencing results as transcriptomic ITH. Hierarchical clustering analysis revealed that most of the expression patterns depend on the interpatient heterogeneity as well as different culture methods. With only a few exceptions, each subregional clone was grouped in accordance with patient origin (**Figure** **3**A). Nevertheless, some subregional clones displayed specifically dispersed patterns. For instance, SNU‐4376AS2‐TO and SNU‐4796S3‐TO subregional clones were clustered separately from their parental origin. In addition, none of the subregional clones of the SNU‐4374‐TO series were clustered at all. PDO subregional clones were evidently separated from PDC subregional clones regardless of spatially diverse clonal capturing (Figure 3A). This indicated that culture conditions had more influence than genetic ITH, and subregional clones cultured in different conditions should not be compared directly by transcriptome analysis.

We also applied our RNA expression data to CMS subtyping by using an R package, CMScaller,^[^ ^28^ ^]^ A total of 78 RNA expression data from the original tumor tissues, PDOs, and PDCs were subtyped (Figure 3B; Table S5A, Supporting Information). We distributed the samples across the subtypes, with the CMS type 2 (*n* = 38) being the most commonly represented. A majority of 12 CRC sets displayed heterogeneous distribution of the CMS types in their derivatives. For instance, all subregional organoids of SNU‐4813 were differently subtyped. Likewise, the SNU‐4376AS2‐TO subregional clone, which was grouped separately in hierarchical clustering, had a distinct subtype in CMS analysis as well. In addition, CMS subtyping indicated that PDOs better recapitulated the original CMS type of the tumor although PDCs retained moderate subregional heterogeneity. Also, two MSI‐H tumors (SNU‐4376A‐TO and SNU‐4398‐TO series) exhibited different CMS types. While SNU‐4398 series were uniformly classified as CMS1 in parallel with its hypermutated and microsatellite unstable nature,^[^ ^29^ ^]^ SNU‐4376A series were subtyped as CMS2 and CMS4.

Next, transcriptomic heterogeneity scores were calculated with the DEPTH algorithm from the R package,^[^ ^30^ ^]^ and considered as the characteristics of each subregional clones, as described by the author (Table S5B, Supporting Information). We drew the box plot to visualize the degree of dispersion of heterogeneity scores within each patient. This indicated the majority of outliers were of 2D cell lines. For instance, SNU‐4351S2 and SNU‐4351S4 had significantly higher DEPTH score than the rest of the subregional clones, which should be considered as transcriptomic variances between 2D and 3D cultures rather than subregional heterogeneity. Besides, the number of derivatives in SNU‐4376Aset (*n* = 8) was higher than other sets due to the presence of 2D cultures, which might put biased weight on the level of heterogeneity. Based on those evidences, we only counted PDOs to determine transcriptomic heterogeneity within a patient. In parallel with previous hierarchical clustering and CMS subtyping analysis, subregional PDOs from SNU‐4374 and SNU‐4376A series were spotted apart from each other (Figure 3C). Also, two MSI‐H tumors (SNU‐4376A and SNU‐4398 series) displayed distinct patterns on DEPTH analysis as well. While the SNU‐4376A series was close to other MSS tumor derivatives, the SNU‐4398 series clustered notably apart from other sets. Also, the degree of intratumor heterogeneity was evidently higher in SNU‐4376A than SNU‐4398.

Overall, these data validated that the subregional PDOs better recapitulate the transcriptomic characteristics of the primary tumor and most of the transcriptomic diversity of CRC than PDCs. It also demonstrates that our multiregional sampling is an effective method for capturing the transcriptomic ITH of the original tumor.

### 2.5 Subregional PDOs Reveal Heterogeneous Drug Responses Caused by ITH

We assembled a 24‐compound library for drug screening. In total, 38 of 43 tumor organoids and 19 of 23 cell lines from the 12 patients were successfully screened in experimental duplicate, generating >1300 measurements of PDO and PDC‐drug interactions. We used complete PDO culture medium when culturing and seeding PDCs for drug treatment in order to minimize the potential variables from different culture conditions.

**Figure 4:**
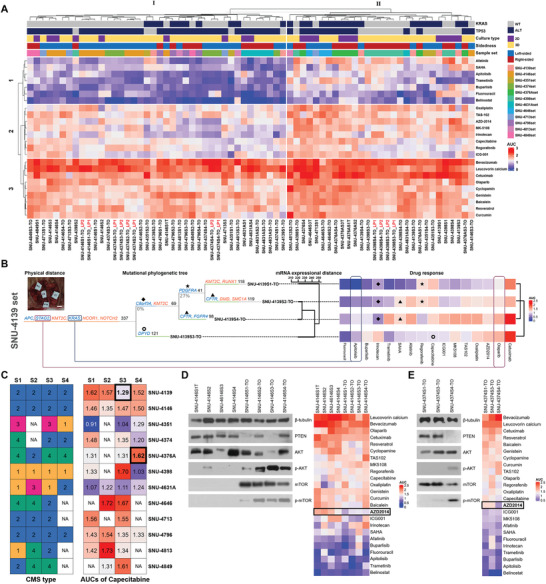
Patient‐derived subregional organoids reveal heterogeneous drug responses caused by molecular ITH. A) See also Table S6A–C in the Supporting Information. Heatmap of AUCs of all 24 compounds against 57 CRC derivatives and 8 low‐passage organoid samples (ending with _LP). The names of drugs are provided on the right. The derivatives and drugs were *k*‐means clustered, based on their AUC values across the drug panel. The Arabic numerals on the left and Roman numerals on the top of the heatmap are assigned to indicate different subgroups according to *k*‐means clustering. The status of *KRAS* and *TP53*, culture types, sidedness, and patient‐origins are displayed above the heatmap. B) See also Figure S6C–K in the Supporting Information. Subregional PDOs reveal heterogeneous drug responses caused by mutational ITH. Mutations observed in the trunk were associated with uniform responses among the subregional clones (marked with red lines, poor response; blue lines, good response, 14 trunk genes observed in 8 sets out of 12). Subregional genetic mutations were associated to heterogeneous drug responses (marked with plane figures, 30 cases observed in 11 sets out of 12). Genes that are listed in the CGC are shown in orange, and genes that are associated with sensitivities to certain drugs are shown in blue on the mutational phylogenetic trees. The Treeomics statistical settings were as follows (sequencing error rate = 0.005, prior absent probability = 0.5, max absent VAF = 0.05, LOH frequency = 0, false discovery rate = 0.05, false‐positive rate = 0.005, and absent classification minimum coverage = 100). Unsupervised Ward's minimum variance method was used as the distance metric for clustering of mRNA expression and drug responses. C) See also Table S6D in the Supporting Information. The transcriptomic CMS subtyping of subregional PDOs enabled the prediction of capecitabine responses. Multivariate analysis of variance (MANOVA) indicated that subregional clones assigned to CMS2 and CMS4 mostly exhibited poorer responses to capecitabine compared to other subtypes (*p* < 0.01). Symbolic colors are used for each CMS subtype (yellow: CMS1; blue: CMS2; red: CMS3; green: CMS4). The names of sample sets are provided on the right. The multifocal numbers (S1–S4) are indicated on the top. Unavailable samples are marked with NA. D) Culture type‐specific protein expression affected the drug response of AZD2014. AKT‐mTOR pathway was specifically activated in only PDOs, which resulted in a better response to AZD2014 compared to PDCs (indicated by bold rectangle). E) Clone‐specific protein expression influenced the drug response of AZD2014. AKT‐mTOR pathway was specifically activated in only a single subregional clone, SNU‐4374S4‐TO, which caused a better response to AZD2014 compared to other subregional clones (indicated by bold rectangle).

As a first validation, the grouping of compounds based on their AUC values confirmed a range of responses across the subregional PDOs and PDCs, and identified five major subgroups in accordance with compounds (**Figure** **4**A). One group (I, 1 coordinate) presented relatively high sensitivity to the EGFR/RAS/RAF/MEK/ERK pathway‐targeting drugs, while the other group (II, 1 coordinate) exhibited heterogeneous sensitivity. Other groups displayed moderate responses to antimetabolites and topoisomerase inhibitors (I, 2 coordinate), or insensitivity (II, 2 coordinate). The final compound set involved phytochemicals, which showed poor responses across all samples (I, 3 and II, 3 coordinates). We mainly focused on the drugs that showed heterogeneous responses across the subregional samples, which includes afatinib, apitolisib, AZD2014, buparlisib, capecitabine, flurouracil, ICG001, irinotecan, MK5108, oxaliplatin, regorafenib, SAHA and trametinib. Importantly, we have included the low‐passage subregional clones (ending with _LP) to assess whether consecutive passaging alters responses to certain drugs. These subregional clones displayed tight correlation to the corresponding high‐passage subregional clones (Figure 4A).

The multifocal samples were likely to be clustered together according to their tumor origin, with variations in several compounds. Within the same cluster, PDCs were grouped adjacent to each other, suggesting that the culture method influences responses to certain drugs. The AUC values calculated from cell lines and organoids were compared using unpaired two‐samples *t*‐test to determine if the variation from the different culture methods had affected the responses of certain drugs. The result indicated multiple drugs including Cetuximab, AZD2014, and Olaparib were affected by the different culture methods (Table S6B, Supporting Information), which indicated the integration of such drug responses of 2D cell lines with other heterogeneity factors such as genetic phylogeny or transcriptomic heterogeneity scores (DEPTH) should be avoided.

Previous studies have demonstrated the sidedness of CRC is correlated with the response of certain chemotherapies.^[^ ^31^ ^]^ We separated the left‐ and right‐sided tumors among our samples, and compared if there are statistically significant variances according to the sidedness. The result revealed the apitolisib and oxaliplatin exhibited better responses in the left‐sided CRCs in our samples in agreement with the earlier research^[^ ^32^ ^]^ (Table S6C and Figure S7A,B, Supporting Information). No correlation between the sidedness and anti‐EGFR therapy was found in our samples.

Then, we integrated the effect of the mutational variance of subregional clones on their heterogeneous responses to various drugs. CGC genes that accounted for the initiation (Figure S6A and Table S4A, Supporting Information) and the evolution (Figure S6B and Table S4B, Supporting Information) in our 12 CRC sets were applied to estimate potential gene‐drug interactions using multivariate analysis of variance (MANOVA). Among eight genes including *TP53* and *KRAS* being statistically significant (Figure 4A), *PABPC1*, *CEP89*, and *RAD51B* exclusively presented in the tumor evolution stage, which implied a possible association with heterogeneous drug responses (Table S6A, Supporting Information). Several studies have demonstrated left‐ and right‐sided colorectal cancers have different genetics and evolutionary trajectories.^[^ ^31^ ^]^ We analyzed the sidedness of MSS tumors separately when evaluating tumor initiating and progressing genes with the following standards. When both number and frequency of the mutation are simultaneously higher in right‐sided tumors than left‐sided tumors, the mutation is assigned to right‐sided mutation. If either the number or the frequency is higher, the mutation is designated to no‐sided mutation. The rest is assigned to left‐sided mutation. The tumor initiating genes (*n* = 78) were subclassified with the left‐sided (*n* = 18), right‐sided (*n* = 48), and no‐sided (*n* = 12) mutations (Figure S6A and Table S4A, Supporting Information). The genes accounting for tumor evolution (*n* = 444) were also subcategorized with the left‐sided (*n* = 156), right‐sided (*n* = 214) and no‐sided (*n* = 74) mutations (Figure S6B and Table S4B, Supporting Information). This analysis revealed that the majority of tumor initiating mutations were of right‐sided, which suggests that right‐sided colorectal tumors had accumulated higher mutational burden within the ancestral clone in our samples than left‐sided tumors. In contrast, genes that are associated with tumor evolution had no disposition toward the sidedness of the tumor.

We further associated the specific mutational aberrations with drug responses by referring previously documented gene‐drug database such as the Drug Gene Interaction Database (DGIdb).^[^ ^33^ ^]^ We specifically focused the subregional clonality supported by the mutational phylogenetic tree and mRNA expressional distances of PDOs since the uneven number of PDCs per each patient depending on the different establishment rates compared to PDOs put biased weight on the level of heterogeneity and possibly shifted the range of AUC values. Thus, for per patient analysis of the drug response, we only included the results from PDOs while PDCs provide sufficient information when integrating entire samples. Genes that are listed in the CGC are shown in orange, and genes that are associated with sensitivities to certain drugs are shown in blue on the mutational phylogenetic trees (Figure 4B; Figure S6C–K, Supporting Information). First, mutations observed in the trunk were associated with uniform responses among the subregional clones (marked with red lines, poor response; blue lines, good response, 14 trunk genes observed in 8 sets out of 12). We also found subregional genetic mutations that are associated to heterogenous drug responses (marked with plane figures, 30 cases observed in 11 sets out of 12). For instance, all subregional clones in SNU‐4139‐TO series harbored an identical *KRAS* mutation (G12V) with VAFs of ≈0.4, and displayed moderately good responses to apitolisib, a dual inhibitor of class I PI3K, in congruence with previous findings.^[^ ^34^ ^]^ Also, a missense mutation at the splice site of *STAG2* (X1193_splice) was observed in all subregional clones of the SNU‐4139‐TO series, and correlated with relatively better responses to Olaparib.^[^ ^35^ ^]^ Among the SNU‐4139‐TO subregional clones, all but SNU‐4139S3‐TO had a mutated *C8orf34* (P59L), which was associated with irinotecan‐derived toxicity^[^ ^36^ ^]^ as the AUC value to irinotecan was significantly higher in SNU‐4139S3‐TO than other subregional clones. In contrast, only SNU‐4139S3‐TO had double mutations at *DPYP* (Arg29Cys and Gln425*), which is related to severe capecitabine toxicity,^[^ ^37^ ^]^ and its AUC value indeed indicated increased toxicity in this specific subregional clone. From this perspective, SNU‐4139S3‐TO subregional clone was independently evolved from the trunk, as indicated by the physical distance on the phylogenetic tree. In addition, both SNU‐4139S2‐TO and SNU‐4139S4‐TO subregional clones contained multiple *CFTR* mutations, which influenced their sensitivities to SAHA.^[^ ^38^ ^]^ Also, *PDGFRA* mutations (D1079N) were developed by SNU‐4139S1‐TO and SNU‐4139S2‐TO subregional clones, which affected their responses to regorafenib^[^ ^39^ ^]^ (Figure 4B). Such analysis for other sets is further depicted in Figure S6C–K in the Supporting Information. Overall, these data suggest subregional mutations substantially affect heterogeneous drug responses within a single tumor.

Next, we associated expression patterns with various drug responses. Since PDOs better retained the primary CMS type of the tumor (Figure 3B) and transcriptional heterogeneity (Figure 3C), we only included PDOs for further evaluation. We assessed the correlation between four CMS types and multiple drug responses using MANOVA. The response of capecitabine (*p* < 0.01) and ICG‐001 (*p* < 0.05) was associated with the CMS types (Table S6D, Supporting Information). For instance, subregional clones assigned to CMS type 2 and 4 generally exhibited poorer responses to capecitabine compared to other subtypes (Figure 4C). Few heterogeneous drug responses within a single set consisting of identical CMS types were explained with the clone‐specific mutations (marked with bold rectangles). For instance, the better response of capecitabine in SNU‐4139S3‐TO was due to the double mutations at *DPYP* (Arg29Cys and Gln425*), which is related to capecitabine toxicity as described above. Also, SNU‐4376AS4‐TO subregional clone harbored a frameshift deletion at *CYP19A1* gene (N486Tfs*5) which is reported to affect the response of capecitabine.^[^ ^40^ ^]^

AZD2014, the reagent inhibiting mTOR activation showed heterogeneous responses across the subregional derivatives (Figure 4A), yet neither genetic aberration nor transcriptional pattern was statistically correlated with its response. For instance, SNU‐4146 series had no mutation in PIK3/AKT/mTOR pathway‐related genes (Figure 2A; Table S2B, Supporting Information), and all derivatives had identical CMS types (Figure 3B). However, distinct separation between PDOs and PDCs was observed in drug responses of the AZD2014 (Figure 4A,D; Table S6B, Supporting Information). Therefore, we further analyzed protein expressions that are related to the molecular target of the AZD2014. The AKT‐mTOR pathway was specifically activated in only PDOs (Figure 4D). This culture type‐specific protein expression resulted in the better responses to AZD2014 to PDOs compared to PDCs in SNU‐4146 series. In addition, a clone‐specific heterogeneity in the protein expression of the AKT‐mTOR pathway was detected in SNU‐4374 series. Only SNU‐4374S4‐TO subregional clone expressed phosphorylated AKT and mTOR, which affected its better response to the AZD2014 (Figure 4E).

### 2.6 Integration of Subregional Transcriptomic and Genomic Analysis Enables Comprehensive Prediction of Heterogeneous Drug Responses

Although the subregional mutations, CMS subtypes, and protein expressions demonstrated the heterogeneous drug responses clone by clone, more comprehensive prediction of subregional drug responses was achieved by integrating genetic and transcriptional variances. As a first validation, the clustering based on the genetic phylogeny and mRNA expression showed comparable patterns, which substantiated that the genetic ITH is well reflected in heterogenous gene expression patterns (marked with dotted lines) (Figure 4B; Figure S6C–K, Supporting Information). We then calculated the Pearson correlation coefficient with *p* values between the transcriptional heterogeneity score (DEPTH) and AUCs of each drug. Among 13 compounds that exhibited heterogeneous drug responses (Figure 4A), the AUC values of eight compounds including afatinib, buparlisib, apitolisib, SAHA, AZD2014, and flurouracil were in direct proportion to the DEPTH score (Table S6E, Supporting Information). All compounds that were in direct proportion to the DEPTH score were of targeted drugs except for flurouracil. This indicates that the elevated expression of oncogenic signatures in high‐ITH tumors diminishes their sensitivity to relevant inhibitors, and the lower expressional deviations from the mean expression values is causative factor for the better response of such targeted drugs.

**Figure 5:**
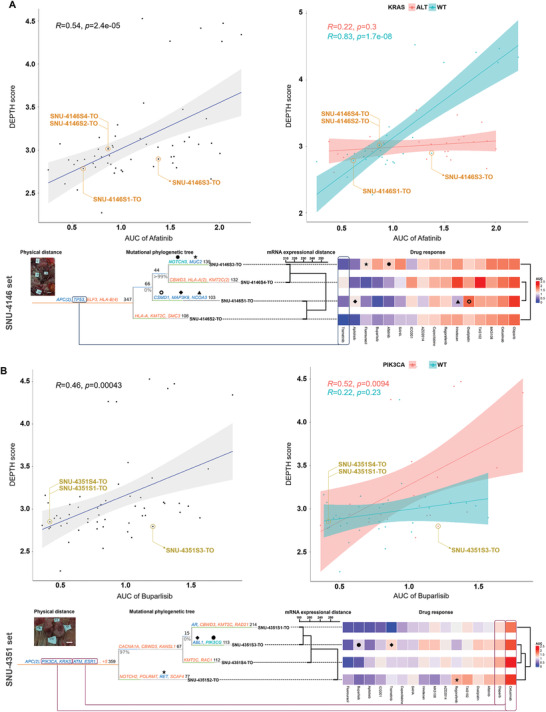
Integration of subregional transcriptomic and genomic analysis enables comprehensive prediction of heterogeneous drug responses. The Pearson correlation coefficient (*R*) with *p*‐values between the transcriptomic ITH score and AUC are indicated on the top of the correlation graph. A value of *p* < 0.05 was considered statistically significant. Confidence intervals are calculated at a confidence level of 0.95 for the parameter and indicated by a shading along with the correlation graph. The specific gene that accounted for unpredicted insensitivity is marked with black circle on the phylogenetic tree. The Treeomics settings were as follows (sequencing error rate = 0.005, prior absent probability = 0.5, max absent VAF = 0.05, LOH frequency = 0, false discovery rate = 0.05, false‐positive rate = 0.005, and absent classification minimum coverage = 100). A) Among the SNU‐4146‐TO series, which consists of four wild type *KRAS* subregional clones, all but SNU‐4146S3‐TO had unpredictable insensitivity by correlation analysis of DEPTH. This subregional clone harbored a unique *NOTCH3* mutation (N1588H), which causes the poor response to afatinib. B) Among the SNU‐4351‐TO series, only the SNU‐4351S3‐TO clone was located outside the confidence interval of the correlation plot. This subregional clone harbored a mutated *PIK3CG*, which is known to cause insensitivity to buparlisib. Unsupervised Ward's minimum variance method was used as the distance metric for clustering of mRNA expression and drug responses.

Drug responses to afatinib had a Pearson correlation coefficient of 0.54 to the DEPTH score (*p* < 0.0005). Since the mutant *KRAS* is already known to cause insensitivity to afatinib, the correlation coefficient was reanalyzed in accordance with the mutational status of *KRAS*. Whereas the altered *KRAS* group displayed no correlation between DEPTH score and afatinib response (*R* = 0.22, *p* > 0.05), the wild type *KRAS* group presented high correlation (*R* = 0.83, *p* < 0.0005) (**Figure** **5**A). Among the SNU‐4146‐TO series, which consists of four *KRAS* wild type subregional clones, all but SNU‐4146S3‐TO were positioned near the confidence interval of the correlation graph, which indicates unpredicted insensitivity of SNU‐4146S3‐TO to afatinib. The DEPTH score alone cannot explain this outlier. However, when genetic mutations were integrated, we found that the SNU‐4146S3‐TO subregional clone harbored a unique *NOTCH3* mutation (N1588H), which has been reported to cause poor response to afatinib (Figure 5A).^[^ ^41^ ^]^ An analogous integration of mutational phylogeny, mRNA expressions, and drug responses also explained the poorer response to buparlisib in SNU‐4351S3‐TO clone (Figure 5B). Among the SNU‐4351‐TO series, only the SNU‐4351S3‐TO clone was outside the confidence interval of the correlation plot. This subregional clone harbored a mutated *PIK3CG* gene, which is known to cause insensitivity to buparlisib (Figure 5B).^[^ ^42^ ^]^

## 3 Conclusion

Patient‐derived tumor organoids have been widely used and represent a promising new platform for personalized cancer medicine,^[^ ^43^ ^]^ reflecting their value for basic cancer studies^[^ ^44^ ^]^ as well as translational research.^[^ ^45^ ^]^ PDO cultures for in vitro modeling of primary tumors have been applied to CRCs as well as other cancer types.^[^ ^17b^ ^]^ CRCs are associated with heterogeneous clones shaped by a Darwinian selection process.^[^ ^13b^ ^]^ The resulting molecular heterogeneity and phenotypic diversity construct a multifaceted clonal architecture, which forms the basis of drug resistance and metastatic potential.^[^ ^46^ ^]^

In this study, we have established 12 sets of CRC PDOs and PDCs in order to capture ITH of individual CRC patients. These models confirmed the highly heterogeneous nature of CRC due to the subregional heterogeneity caused by the parallel genetic and transcriptional evolution within a single tumor. Our genetic phylogeny analysis revealed many of the known cancer mutations (e.g., the Vogelgram genes: *APC*, *KRAS*, *SMAD4*, *TP53*) were found to be shared in the trunk of individual subregional clones, but were rarely specific to sublineages, which suggests that the genetic ITH is probably driven by less characterized cancer mutations. Since we have mixed the DNA from each region of a single tumor, we were not able to construct the phylogenetic trees directly from the tissue, which restricted the valuation of the extent to which the subregional organoid recapitulates the genetic of the subregional tissue. Our work also confirmed that both PDOs and PDCs comparably retain the genomic heterogeneity of primary tumors, yet PDOs are superior preclinical model to PDCs in perspective of capturing the transcriptomic and proteomic heterogeneity. In addition, our multiregional models retaining the molecular ITH from primary tumors enabled the integration of highly prevalent genetic and transcriptional heterogeneity to corresponding heterogeneity of drug responses in CRC.

Taken together, our approach provides a high‐fidelity preclinical cancer model to understand the evolutionary trajectories of individual tumors. By capturing the hierarchical clonal structure with live sets of PDOs and PDCs, which recapitulate the histopathological and molecular heterogeneity of human CRC, our data revealed heterogeneous drug responses of CRC in the perspective of the prevalent molecular heterogeneities, and provide potential resolutions to overcome the drug resistance caused by ITH using various molecular layers. It also suggests that single biopsy‐based PDOs have intrinsic limitations for predicting patient responses. As a potential solution, our data show the importance of targeting ancestral somatic driver mutations shared by cancer subregional clones in conjunction with comprehensive understandings of transcriptomic and proteomic tumor heterogeneity in order to defy wanted drug resistance caused by ITH.

## 4 Experimental Section

### Nomenclature

 
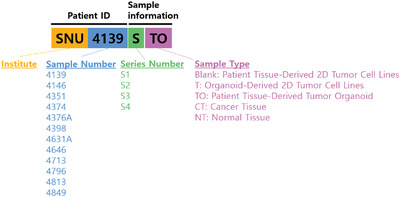


### Data and Material Availability

Raw and processed next‐generation sequencing data will be available by request and governed by Ja‐Lok Ku. Computational pipelines in this study are available at the public repository (https://github.com/mario2437/CRC_HETERO). Cell lines and organoids generated in this study have been deposited to the KCLB (Korean Cell Line Bank, https://cellbank.snu.ac.kr) biobank and are governed by Ja‐Lok Ku.

### Ethics Statement

The research protocol was reviewed and approved by the institutional review board of the Seoul National University Hospital (IRB No. 1102‐098‐357). The study was performed in accordance with the Declaration of Helsinki. Written informed consent was obtained from all patients enrolled in this study.

### Sample Collection and Preparation

A total of 45 multifocal specimens of human colorectal tumors were collected from 12 CRC patients who underwent radical resection at Seoul National University Hospital (Seoul, Korea). Tumor tissues were histologically diagnosed by a pathologist as adenocarcinoma. The surgically resected tumor tissue was submerged in Opti‐MEM supplemented with 1% penicillin/streptomycin (GIBCO, CA, USA, Cat#15140‐122) and transferred in an ice box directly from the operating room to the laboratory. The resected colon tissue was laterally unfolded in order to expose the neoplasia gland. The multifocal regions of the primary tumor were designated as S1–S4 before preprocessing for the further culture. The minimum lateral distance between two multisite samples was 1 cm. The size of the tissue fragment for extracting DNA and RNA was set to 0.5–0.8 cm^3^. When the size of the tumor mass did not meet the criteria, the samples were excluded from the multisampling analysis. The distance between the different multifocal sites is given in **Table** **2**.

**Table 2:** The distance between the different multifocal sites (unit: cm)

	SNU‐4139	SNU‐4146	SNU‐4351	SNU‐4374	SNU‐4376A	SNU‐4398
S1–S2	4.56	11.21	5.79	3.69	10.96	4.87
S1–S3	2.25	4.96	2.76	2.21	5.51	2.61
S1–S4	4.56	5.42	4.09	2.20	12.09	5.13
S2–S3	2.22	6.35	3.87	1.74	5.62	3.39
S2–S4	4.62	7.47	3.61	3.11	5.81	3.82
S3–S4	2.49	3.56	4.40	2.08	7.40	5.28

	SNU‐4631A	SNU‐4646	SNU‐4713	SNU‐4796	SNU‐4813	SNU‐4849
S1–S2	5.45	5.70	2.64	4.67	5.50	2.98
S1–S3	3.55	3.31	3.26	2.40	2.95	3.82
S1–S4	5.88	NA	NA	2.43	7.06	NA
S2–S3	3.18	2.79	2.98	2.59	3.87	3.62
S2–S4	3.06	NA	NA	3.61	5.08	NA
S3–S4	2.65	NA	NA	1.67	4.13	NA

Detailed information about tumor sizes and locations is summarized in Table 1. The starting material information is indicated by the last characters of the sample names: Blanks, patient‐derived 2D tumor cell lines; T, organoid‐derived 2D tumor cell lines; TO, patient‐derived tumor organoid; NT, normal tissue; CT, cancer tissue. Genomic DNA was extracted from resected tumor tissues and paired adjacent normal mucosa using the All Prep DNA/RNA Mini Kit (Qiagen, Hiden, Germany) according to manufacturers’ protocol.

### Tumor Isolation and Culture

Once the multiregional sites were labeled, primary tumor tissues were photographed and washed in cold PBS with penicillin/streptomycin (GIBCO, Cat#15140‐122) five times. In accordance with the labels (S1–S4), tumor tissues were cut into three to four pieces. Both DNA and RNA were extracted from 0.5 to 0.8 cm^3^ of tissue materials from each multifocal region. The weight per each tissue material was set to 8–10 mg. Then, the DNA and RNA fragments from each site were mixed with strictly measured quantities (50 µL of 300 ng µL^−1^ solution per each site). For immunohistochemistry, the middle portion of the tumor tissue was used. Cell lines were established using a previously published protocol.^[^ ^46^ ^]^ For the organoid cultures, the remaining tumor tissue from each site was minced into smaller fragments on a sterilized dish on ice and incubated with digestion (5–10 mL) medium supplemented with collagenase II (1.5 mg mL^−1^) (GIBCO, Cat# 17101‐015), hyaluronidase (20 µg mL^−1^) (Sigma‐Aldrich, MO, USA, Cat# H3506) and Ly27632 (10 × 10^−6^ m) (Selleckchem, TX, USA, Cat# S1049) for 30 – 60 min depending on the size of tumor piece at 37 °C on a shaker. The mixture was applied to a 100 µm cell strainer (SPL Life Sciences, Gyeonggi‐do, Korea, Cat# 93100) to remove large fragments. Tumor cells were centrifuged at 1000 rpm for 3 min, and resuspended in basal culture medium (Dulbecco's modified Eagle medium/F12, 1% penicillin/streptomycin, Glutamax (10 × 10^−3^ m), 10% FBS). This process was repeated twice to remove debris and digestion solution. Then, red blood cell (RBC) lysis buffer (2–5 mL) (Sigma‐Aldrich, cat# R7757) was added for 1–5 min to remove RBCs. Tumor cells were spun down at 1000 rpm for 3 min, and resuspended in BME (GIBCO, Cat# A14132‐02) for seeding in a T‐25 flask (Corning, NY, USA, Cat# 353108). Approximately 17–19 BME (Basement Membrane Extracts) domes (each consisting of BME (50 µL) containing 20 000 cells mL^−1^) were seeded in a single T‐25 flask. The flask was incubated at 37 °C for 10 min. Once BME had solidified, 5 mL of human intestinal stem cell (HISC) medium was added, and cells were incubated in a 37 °C and 5% CO_2_ culture incubator. The HISC medium consisted of 40% W/V basal culture medium, 50% W/V L‐WRN conditioned medium, 1× B27 (GIBCO, Cat# 17504‐044), human EGF (50 ng mL^−1^) (GIBCO, Cat# PHG0313), human FGF‐10 (10 ng mL^−1^) (Peprotech, Cat# 100–26), nicotinamide (10 × 10^−3^ m) (Sigma‐Aldrich, Cat# 72340), *N*‐acetylcysteine (1.25 × 10^−3^ m) (Sigma‐Aldrich, Cat# A7250), A83‐01 (500 × 10^−9^ m) (Sigma‐Aldrich, Cat# SML0788), SB202190 (3 × 10^−6^ m) (Sigma‐Aldrich, Cat# S7067), prostaglandin E_2_ (Sigma‐Aldrich, Cat# P5640), and primocin (100 µg mL^−1^) (GIBCO, Cat# ant‐pm‐1).

### Colorectal Cancer Organoid Cultures

Human CRC organoid culture medium was refreshed every three days. CRC organoids were photographed at initial passages (p1–p5). For passaging, the BME dome was mechanically pipetted using TrypLE Express solution (GIBCO, Cat# 12604‐021) and organoids were collected in a 15 mL conical tube. The BME dome was mechanically dissociated with intense pipetting, and the tube containing the organoids and BME mixture was incubated at 37 °C for ≈5–10 min. The organoids were centrifuged at 1000 rpm for 3 min, and the supernatant was aspirated. Once BME was removed, the cell pellet was resuspended with fresh BME, seeded in a T‐25 flask and the flask was incubated at 37 °C for 10 min. Once BME was solidified, HISC medium (5 mL) was added to the flask to overlay the BME dome and cells were incubated in a 37 °C and 5% CO_2_ culture incubator.

### Cell Line and Organoid Seeding/Drug Treatment Procedure

All drug screens were repeated twice. For cell lines, depending on various growth rates, 2–8 × 10^5^ cells mL^−1^ were seeded in clear‐bottomed, white‐walled 96‐well plates (Corning, Cat# 3903) in complete organoid culture medium (80 µL) in order to minimize the potential variables from different medium conditions that contain fetal calf serum (FCS) and incubated in humidified incubators at 37 °C for 24 h in an atmosphere of 5% CO_2_. Organoids were enzymatically and mechanically dissociated into single cells by incubating and pipetting in TrypLE Express solution (GIBCO) for 5 to 10 min. The mixture was spun down at 1500 rpm for 3 min. Once BME was removed, the cell pellet was resuspended with 1:1 mixture of HISC medium and BME (GIBCO). Homogenous BME mixture (40 µL, 20 000 organoids mL^−1^) was plated in clear‐bottomed, white‐walled 96‐well plates (Corning, Cat# 3903) using E1‐ClipTip Electronic Pipettes (Thermo Fisher Scientific, MA, USA). Plates were incubated at 37 °C with 5% CO_2_ for 15 min to solidify the BME before adding prewarmed HISC medium (40 µL) to each well using an EpMotion 5070 (Eppendorf Corporate, Hamburg, Germany). 96 h after seeding, drug solution (20 µL) was added to each well. Drugs were serially diluted in DPBS at a ratio of 1:3 from the maximum dose to make 6 dose points. For the control well, the mixture of HISC medium and drug‐solvent solution was added. Detailed information about compounds is given in **Table** **3**.

**Table 3:** Detailed information about compounds

Drugs	Company	Cat No.	Stock quantities [mg]	Solvent	Max concentration [µm]
5‐FU	Sigma‐Aldrich	Cat# S1209	200	DMSO	20 000
Afatinib (BIBW2992)	Selleckchem	Cat# S1011	10	DMSO	50
Apitolisib (GDC0980)	Selleckchem	Cat# S2696	10	DMSO	50
Avastin (Bevacizumab)	Selleckchem	Cat# A2006	5	DMSO	1000 µg mL^−1^
Baicalein	Selleckchem	Cat# S2268	50	DMSO	100
Belinostat (PXD101)	Selleckchem	Cat# S1085	10	DMSO	100
Buparlisib (BKM120)	Selleckchem	Cat# S2247	10	DMSO	100
Camptosar (Irinotecan Hydrochloride)	Selleckchem	Cat# S2217	25	DMSO	100
Curcumin	Selleckchem	Cat# S1848	50	DMSO	100
Cyclopamine	Selleckchem	Cat# S1146	10	EtOH	50
Eloxatin (Oxaliplatin)	Sigma‐Aldrich	Cat# S1224	50	DMSO	100
Erbitux (Cetuximab)	Selleckchem	Cat# A2000	5	DMSO	1000 µg mL^−1^
Genistein	Selleckchem	Cat# S1342	100	DMSO	100
ICG‐001	Selleckchem	Cat# S2662	25	DMSO	100
Lonsurf (TAS‐102)	Selleckchem	Cat# S8539	25	DMSO	100
MK‐5108	Selleckchem	Cat# S2770	10	DMSO	100
Olaparib (LYNPARZA)	Selleckchem	Cat# S1060	25	DMSO	50
Resveratrol	Selleckchem	Cat# S1396	100	DMSO	200
Stivarga (Regorafenib)	Selleckchem	Cat# S1178	10	DMSO	100
Trametinib (GSK1120212)	Selleckchem	Cat# S2673	10	DMSO	50
Vistusertib (AZD2014)	Selleckchem	Cat# S2783	10	DMSO	5
Vorinostat (SAHA)	Selleckchem	Cat# S1047	200	DMSO	50
Wellcovorin (Leucovorin Calcium)	Selleckchem	Cat# S1236	50	ddH2O	10 000
Xeloda (Capecitabine)	Selleckchem	Cat# S1156	50	DMSO	1000

### Adenosine Triphosphate Assay

After 72 h of drug treatment, CellTiter‐Glo (10 µL) (Promega, Cat# G7572) was added to each well for cell lines and CellTiter‐Glo 3D Reagent (10 µL) (Promega Cat# G968B) was added to each well for organoids, followed by 5 min of vigorous shaking. After 30 min incubation at room temperature and an additional minute of shaking, luminescence was measured with a Luminoskan Ascent (Thermo Fisher Scientific) over 1000 ms of integration time. Data were normalized to vehicle and area under curve (AUC) values were calculated using R program version 3.6.3 (R Foundation for Statistical Computing, Vienna, Austria).

### Western Blot Analysis

For cell lines, cells were harvested with a cell scraper after washing with cold PBS three times. For organoids, HISC medium was aspirated and BME dome was washed with cold PBS three times. Then, BME dome was mechanically pipetted using cell recovery solution (5–10 mL) for 2–3 h on ice while shaking and organoids were collected in a tube. Cell pellets were washed with cold PBS three times. Whole protein was extracted with EzRIPA buffer (ATTO Co., Tokyo, Japan) supplied with 1% protease inhibitor (ATTO Co.) and 1% phosphatase inhibitor (ATTO Co.). The volume of lysis buffer was adjusted to the number of cells collected in each vial. The protein concentration was determined by Pierce BCA Protein Assay Kit (Thermo Fisher Scientific). Mixture of equal amounts of protein, SDS buffer (Invitrogen, CA, USA), reducing buffer (Invitrogen), and distilled water was boiled at 98 °C for 10 min. Then, the mixture was loaded on 4–15% Mini‐PROTEAN TGX Precast Gels (BIO‐RAD, CA, USA) and blotted at 50 V for 2 h. Proteins were then transferred to Trans‐Blot Turbo Transfer Pack (BIO‐RAD) using Trans‐Blot Turbo Transfer System V1.02 machine (BIO‐RAD) at 2.5 Amp and 25 V. The membrane was incubated in 2.5% skim milk (BD biosciences, NJ, USA) containing 1 × 10^−3^ m MgCl2, 10% TBS buffer, and 0.5% Tween 20 (VWR Life Science, PA, USA) for an hour at room temperature. Primary antibodies and the dilution factors used in this study are summarized in **Table** **4**.

**Table 4:** Primary antibodies and the dilution factors used in this study

Target	Company	Cat No.	Dilution
Rabbit monoclonal anti‐mTOR	Cell Signaling Technology	Cat# 2983; RRID:AB_2105622	1:1000
Rabbit monoclonal anti‐pmTOR (Ser2448)	Cell Signaling Technology	Cat# 2971; RRID:AB_330970	1:1000
Mouse monoclonal anti‐panAKT	Cell Signaling Technology	Cat# 2920; RRID:AB_1147620	1:1000
Rabbit monoclonal anti‐pAKT (Thr308)	Cell Signaling Technology	Cat# 86758; RRID:AB_2800089	1:1000
Rabbit polyclonal anti‐PTEN	Cell Signaling Technology	Cat# 9552; RRID:AB_10694066	1:1000
Rabbit polyclonal anti‐*β*‐tubulin	Cell Signaling Technology	Cat# 2146; RRID:AB_2210545	1:2000

After washing with 2.5% skim milk three times, mouse or rabbit IgG secondary antibody (Jackson ImmunoResearch Labs, PA, USA) conjugated with peroxidase diluted in 2.5% skim milk solution (1:5000) was applied to membrane. SuperSignal West Pico PLUS Chemiluminescent Substrate (Thermo Fisher Scientific) was applied to the membrane which was then exposed to Fuji RX film (Fujifilm, Tokyo, Japan) for 1–10 min.

### H&E Staining and Immunohistochemistry

Tumor tissues were fixed in 10% neutral buffered formalin and embedded in paraffin. Then, tissues were sectioned at 4 µm thickness. For organoids, the BME dome was mechanically scraped with a pipet tip. Cold PBS (10 mL) was added to collect dissociated BME domes and transferred to a 15 mL conical tube. After 15 s centrifugation at 100 rpm, the supernatant was aspirated. This procedure was repeated until the BME gel was visibly removed. Care was taken not to destroy the original structure of the organoids. Collected organoids were embedded in 2% agarose gel (INTRON Biotechnology, Seongnam, Korea). Solidified agarose gel was fixed in 10% formalin for 30 min at room temperature and sectioned at 4 µm thickness. Sections were subjected to H&E as well as immunohistochemical staining. For immunohistochemistry, the Ventana BenchMark XT Staining system (Ventana Medical Systems, AZ, USA) and OptiView universal DAB kit (Ventana, Cat# 760‐700) were used according to the manufacturers’ protocol. Briefly, the sectioned tissues or organoids were mounted on a positively charged glass microscope slide. Slides containing the section were baked for 2 h in a 60 °C oven. Then, the slides were deparaffinized with EZ prep solution (Ventana) at 76 °C for 4 min. To increase renaturation of protein molecules and antibody accessibility, pH 8.4 cell conditioning 1 (CC1) buffer (Ventana) was applied to the slide at 100 °C for 24 min followed by 3% H_2_O_2_ solution treatment at 37 °C for 4 min. Primary antibodies were applied at 37 °C for 16 min. Antibodies used for immunohistochemistry are summarized in **Table** **5**.

**Table 5:** Antibodies used for immunohistochemistry

Target	Company	Catalog	Dilution
Mouse monoclonal anti‐Cytokeratin 20	Santa Cruz Biotechnology	Cat# sc‐271183; RRID:AB_10610054	1:200
Mouse monoclonal anti‐CDX2	BioGenex	Cat# AM392; RRID:AB_2650531	1:300
Mouse monoclonal anti‐*β*‐catenin	BD Biosciences	Cat# 610153; RRID:AB_397554	1:800
Rabbit polyclonal anti‐KI‐67	Abcam	Cat# ab15580; RRID:AB_443209	1:500
Mouse monoclonal anti‐MLH1	Santa Cruz Biotechnology	Cat# sc‐56161; RRID:AB_784582	1:500
Mouse monoclonal anti‐MSH2	Santa Cruz Biotechnology	Cat# sc‐137015; RRID:AB_2144968	1:500
Mouse monoclonal anti‐MSH6	Santa Cruz Biotechnology	Cat# sc‐271080; RRID:AB_10611658	1:500
Mouse monoclonal anti‐PMS2	Santa Cruz Biotechnology	Cat# sc‐25315; RRID:AB_628163	1:500

HQ linker and HRP multimer solution (Ventana) was applied at 37 °C for 8 min. Then, 3,3′‐diaminobenzidine tetrahydrochloride (DAB) chromogen (Ventana) was applied at 37 °C for 8 min followed by hematoxylin and bluing reagent treatment at 37 °C for 8 min. The stained positive tissue control was examined with hematoxylin to ascertain that all reagents were functioning properly.

### Immunocytochemistry

The BME dome was mechanically scraped with a pipet tip. Cold PBS (10 mL) was added to collect dissociated BME domes and transferred to a 15 mL conical tube. After 100 rpm, 15 s centrifugation, the supernatant was aspirated. This procedure was repeated until the BME gel was visibly removed. Care was taken not to destroy the original structure of the organoids. Then, organoids were fixed and permeabilized with BD Cytofix/Cytoperm (BD Science, CA, USA). After cells were washed with washing solution (BD Science), DPBS containing 2% FBS (GE Healthcare Life Sciences, Buckinghamshire, UK) was applied for an hour. After organoids were washed with cold DPBS, CD133 antibody (Abcam, Cambridge, UK) (1:400) diluted in 0.05% of PBS.T was applied for 1.5 h at room temperature. Thereafter, cells were washed with 0.05% of PBS.T, and Alexa 488 and Alexa 568 secondary antibodies (Thermo Fisher Scientific, 1:500) diluted in 0.05% of PBS.T were applied for an hour at room temperature. DAPI (1:100) and rhodamine‐conjugated phalloidin (Sigma‐Aldrich, 1:10) were diluted in distilled water and applied for 30 min at room temperature. Cells were washed with DPBS three times and placed under a confocal microscope. LSM800 Confocal Laser Scanning Microscope and ZEN software (Carl Zeiss, Oberkochen, Germany) were used to analyze images. Digital resolution, scan speed, and the number of pictures averaged were set to 1024 × 1024, 40 s per one channel, and 8 pictures, respectively. Diverse magnifications were used in accordance with growth patterns and sizes of cells. The intensity of each channel was fixed for comparing target protein expression between samples.

### Whole‐Exome Sequencing

Whole‐exome capture was performed on all samples with the SureSelect Human All Exon V5 Kit (Agilent Technologies, Tokyo, Japan). The captured targets were subjected to sequencing using HiSeq 2500 (Illumina, San Diego, CA, USA) with 100× coverage depth for the DNA extracted from organoids and cell lines, and 200× coverage depth for the DNA extracted from the tissue samples. The sequence data were processed through an in‐house pipeline. Briefly, paired‐end sequences were first mapped to the human genome, where the reference sequence was UCSC assembly hg19 (original GRCh37 from NCBI, Feb. 2009) using the mapping program BWA (version 0.7.12), and generated a mapping result file in BAM format using BWA‐MEM. Then, Picard‐tools (ver.1.130) were applied in order to remove PCR duplicates. The local realignment process was performed to locally realign reads with BAM files reducing those reads identically matched to a position at the start into a single one, using MarkDuplicates.jar, which required reads to be sorted. By using the Genome Analysis Toolkit, base quality score recalibration (BQSR) and local realignment around indels were performed. Haplotype Caller of GATK (GATKv3.4.0) was used for variant genotyping of each sample based on the BAM file previously generated (SNP and short indel candidates were detected). Somatic mutations were identified by providing the reference and sequence alignment data of tumor tissues or organoids to the MuTect2 (involved in GATK v3.8.0) with default parameters using tumor‐normal mode. The matched normal tissue was not available for SNU‐4376 series, and peripheral blood mononuclear cells (PBMCs) were used for somatic mutation calling. Those variants were annotated by SnpEff v4.1g to vcf file format, filtering with dbSNP for the version of 142 and SNPs from the 1000 Genome Project. Then, SnpEff was applied to filter additional databases, including ESP6500, ClinVar, dbNSFP 2.9. Mutational signatures were evaluated using the Mutational Patterns R package, release 3.6.1^[^ ^47^ ^]^ to configure distinct footprints in genomic context for all somatic SNVs and evaluate a multitude of mutational patterns of base substitution in tumor tissues and matched cell lines/organoids.

### Analysis of CNVs

For the detection of CNVs and LOH from exome sequencing data, the ExomeCNV package was employed in the R program.^[^ ^48^ ^]^ The final log ratio of depth of coverage was determined by the number of bases targeted by exome sequencing (targeted base) and the number of bases actually sequenced (mapped). CNV calls were expressed as 1, 2, and 3, which indicated deletion, normal, and amplification, respectively.

### Construction of Evolutionary Trees

The evolutionary trajectories of twelve CRC cases were traced using the Treeomics algorithm^[^ ^26^ ^]^ and WES data of multiregion samples. The Treeomics settings were identical for all samples (sequencing error rate = 0.005, prior absent probability = 0.5, max absent VAF = 0.05, LOH frequency = 0, false discovery rate (FDR) = 0.05, false‐positive rate = 0.005, and absent classification minimum coverage: 100). Input parameters included read depths of both mutant and coverage genes, gene symbols, chromosomal coordinates, and substitutional patterns. The Treeomics algorithm also provided likely driver gene mutations and built‐in Cancer Gene Census list. Although the *MUC6* gene was included in Cancer Gene Census genes, the number of mutations in the *MUC6* gene outnumbered other genes. Consequently, the structure of the evolutionary trees was largely affected by the *MUC6* variations. To eliminate potential bias, mutations of the *MUC6* gene were excluded from the input data. Sequencing artifacts were automatically adjusted by the Treeomics default setting to confirm the topologic configuration of the evolutionary tree was compatible with the mutational patterns. Subregional clone analysis was conducted by adding “‐u” parameter to input commands.

### Analysis of RNA Sequencing

Total RNA was isolated from cell lysate using TRIzol (Qiagen) and Qiagen RNeasy kit (Qiagen, Cat# 74104). Paired end sequencing reads of cDNA libraries (101 bp) generated from a NovaSeq6000 instrument were verified with FastQC v 0.11.7. For data preprocessing, low quality bases and adapter sequences in reads were trimmed using Trimmomatic v0.38. The trimmed reads were aligned to the human genome (UCSC hg19) using HISAT v2.1.0, a splice‐aware aligner. Then, transcript assembly of known transcripts, novel transcripts, and alternative splicing transcripts was processed by StringTie v1.3.4d.^[^ ^49^ ^]^ Based on the result, expression abundance of transcript and gene were calculated as read count or FPKM value (fragments per kilobase of exon per million fragments mapped) per sample. Consensus molecular subtype (CMS) of each sample was analyzed by R package, CMScaller.^[^ ^29^ ^]^ Raw readcounts were used as a direct input with “RNA‐seq = True” setting. Heterogeneity scores were calculated with the DEPTH algorithm from the R package^[^ ^31^ ^]^ with default setting. CeL‐ID package^[^ ^50^ ^]^ was also applied for authentication of lines. FASTQ files were processed from the RNA‐seq to call the variants, which provided sequencing depth and allele frequency information. Briefly, the trimmed reads using Trimmomatic v0.38 were aligned to the human genome (UCSC hg19) using STAR 2.6.0c. Then, GATK pipeline v.4.2.0.0 including STAR 2‐pass mapping, Picard MarkDuplicate, Split “N” Trim, Realignment, and Base recalibration steps was applied. Haplotype Caller was used for variant genotyping of each sample based on the BAM file previously generated.

### Statistical Analysis

For hierarchical cluster analysis on a set of dissimilarities, each object was assigned to its own cluster, which an algorithm proceeds through iteratively. Two of the most similar clusters were joined at each stage until there was a single cluster. Distances between clusters were recomputed at each stage by the Lance–Williams dissimilarity update formula according to the particular clustering method being used. Clustering methods included: Ward's minimum variance method, *k*‐means method, complete linkage method, and single linkage method. For the CMS analysis, readcounts were used from the RNA‐seq data as indicated from the R package, CMScaller.^[^ ^29^ ^]^ The data were presented with *p*‐values with FDR. The heterogeneity score was presented with box plot indicating minimum score, first (lower) quartile, median, third (upper) quartile, and maximum score. Outliers from the heterogeneity score were kept due to the transcriptomic variance between 2D and 3D cultures. A multivariate analysis of variance (MANOVA) model was applied to the drug response data matrix with various factors including the mutational status and CMS subtypes. The mutational status of 20 selected genes were tested to AUC values across 24 drugs (*n* = 57). The data was presented with Pillai's trace score, approximate F value and *p*‐value for each of the gene–drug pairs. The four CMS subtypes were tested to AUC values across 24 drugs as well (*n* = 57). The data were presented with sum of squares, mean square, *F* value, and *p*‐value for each drug. Unpaired two‐samples *t*‐test was performed in order to determine compounds in which its AUC was affected by the two different cultures types (2D vs 3D) and the sidedness of the tumor (left‐ vs right‐sided) (*n* = 69). The result was presented with *p*‐value, confidence interval, estimate mean, and standard error for each drug. A value of *p* < 0.1 was considered statistically significant. Outliers from the drug screening results were kept since it can be derived from the insensitivity for specific drug. The linear dependence between DEPTH scores and AUC values for 24 drugs was calculated with Pearson correlation (*n* = 57). Confidence intervals were calculated at a confidence level of 0.95 for the parameter. A value of *p* < 0.05 was considered statistically significant unless otherwise specified. All statistical analysis was performed in R program version 4.0.4 unless otherwise specified.

## Conflict of Interest

The authors declare no conflict of interest.

## Author Contributions

S.‐C.K., S.‐Y.J., and J.‐L.K. conceptualized the paper. S.‐C.K., J.W.P., M.J.K., S.‐Y.J., and J.‐L.K. prepared the methodology and S.‐C.K. worked with the software. S.‐C.K. and H.‐Y.S. were involved in validation and visualization. S.‐C.K. carried out formal analysis and data curation. Investigation was carried out by S.‐C.K., H.‐Y.S., J.‐H.P., G.‐H.K., and J.O.L. J.W.P., H.‐Y.S., M.J.K., J.‐H.P., G.‐H.K., J.O.L., Y.‐K.S., J.M.B, S.‐Y.J., and J.‐L.K. worked with the resources. S.‐C.K. prepared the original draft. S.‐C.K., J.W.P., B.‐K.K., S.‐Y.J., and J.‐L.K. were involved in writing, review, and editing. J.W.P., B.‐K.K., S.‐Y.J., and J.‐L.K. did the supervision. S.‐Y.J. and J.‐L.K. were involved in project administration. S.‐C.K., Y.‐K.S., and J.‐L.K were involved in funding acquisition.

## Supporting information

Supporting Information

Supplemental Table 1

Supplemental Table 2

Supplemental Table 3

Supplemental Table 4

Supplemental Table 5

Supplemental Table 6

## Data Availability

The data that support the findings of this study are available from the corresponding author upon reasonable request.
