# Supplementary material for: Multifocal Organoid Capturing of Colon Cancer Reveals Pervasive Intratumoral Heterogenous Drug Responses
Source: Adv Sci (Weinh). 2021 Dec 17;9(5):2103360. doi: 10.1002/advs.202103360 (PMC8844556; doi:10.1002/advs.202103360)
Supplement: Supplementary file 1 — Supporting Information [file ADVS-9-2103360-s004.pdf]

ADVANCED SCIENCE
Open Access

Supporting Information

for *Adv. Sci.*, DOI: 10.1002/advs.202103360

Multifocal Organoid Capturing of Colon Cancer Reveals Pervasive Intratumoral Heterogenous Drug Responses

Soon-Chan Kim, Ji Won Park, Ha-Young Seo, Minjung Kim, Jae-Hyeon Park, Ga-Hye Kim, Ja Oh Lee[1,3], Young-Kyoung Shin, Jeong Mo Bae, Bon-Kyoung Koo, Seung-Yong Jeong[*], and Ja-Lok Ku[*]

Supporting Information

Multifocal Organoid Capturing of Colon Cancer Reveals Pervasive Intratumoral Heterogenous Drug Responses

Soon-Chan Kim, Ji Won Park, Ha-Young Seo, Minjung Kim, Jae-Hyeon Park, Ga-Hye Kim, Ja Oh Lee, Young-Kyoung Shin, Jeong Mo Bae, Bon-Kyoung Koo, Seung-Yong Jeong*, and Ja-Lok Ku*

Figure S1

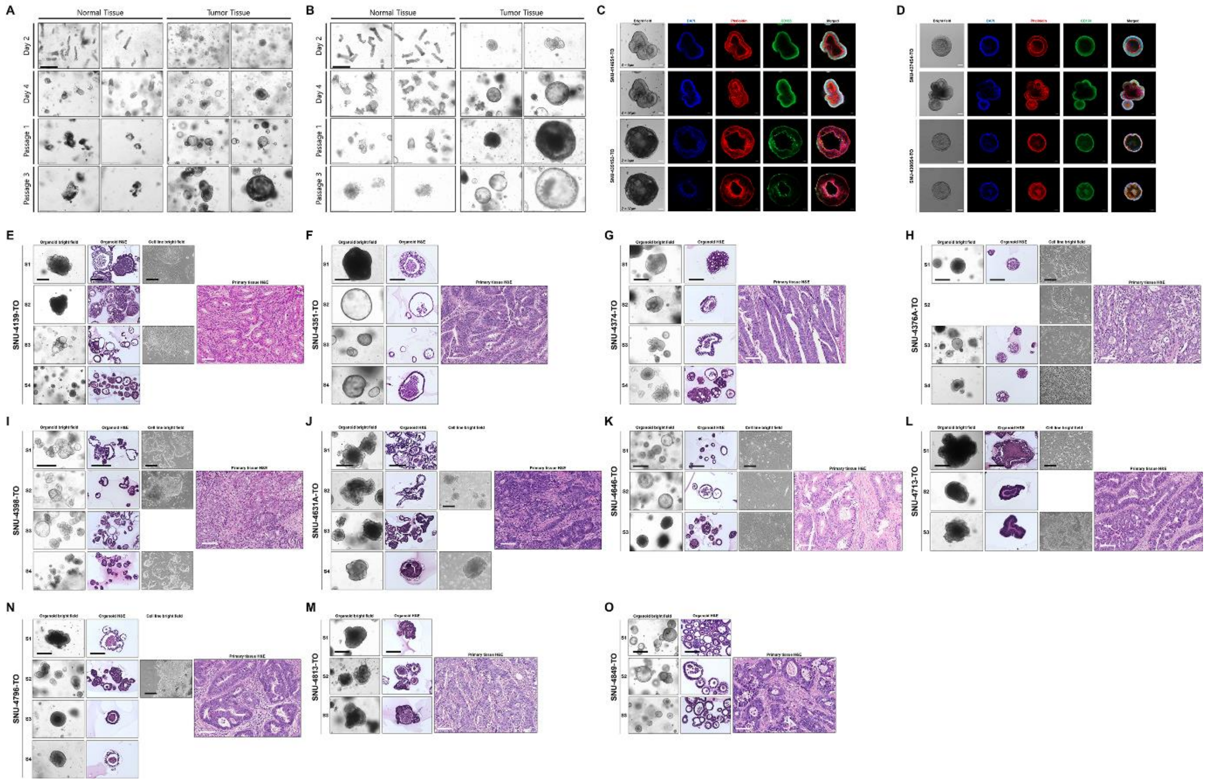

Figure S1A,B. Expansion of normal organoids ceases completely in tumor organoid medium. The structure of normal crypts started to be dissociated within three days and cryptic architecture was completely destroyed after passaging. Scale bars = 500 µM. **Figure S1C,D.** Immunocytochemistry of patient-derived organoids (*n* = 4). Scale bars = 500 µM. **Figure S1E-O.** Histopathological characterization of patient-derived cell lines (*n* = 21), organoids (*n* = 43) and tissues (*n* = 12). Scale bars = 500 µM.

Figure S2

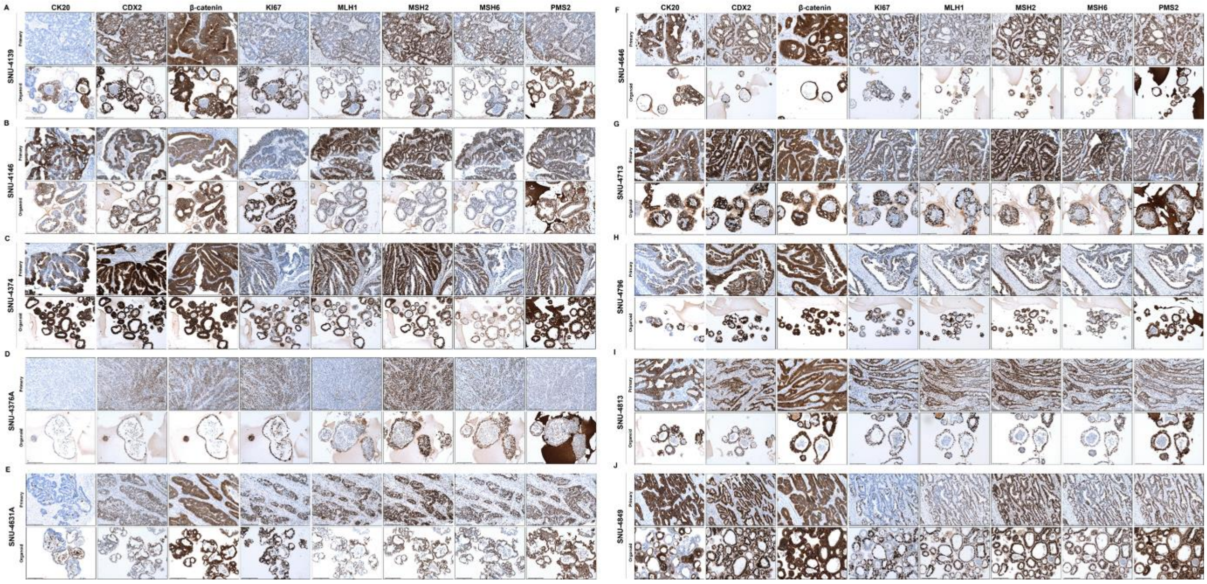

Figure S2. A-J. Immunohistochemistry of patient-derived organoids. Scale bar = 200 μM.

Figure S3

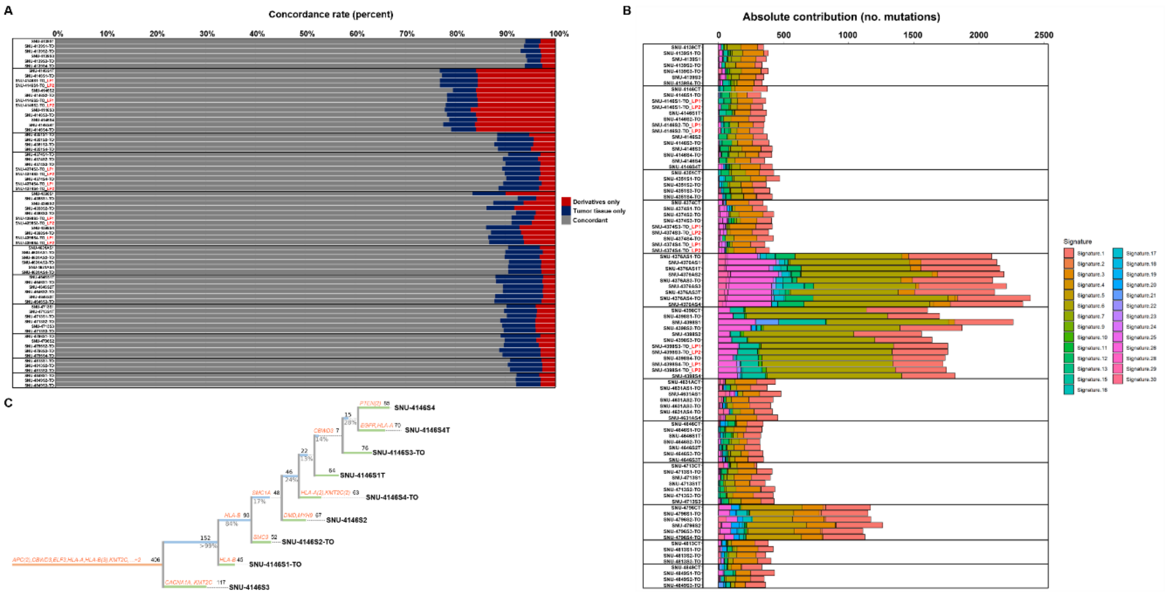

Figure S3A. See also Table S3A. Histogram showing the concordance (percent) of mutations between derivatives (*n* = 71) and corresponding primary tumors. Mutational concordances within the coding regions corresponded closely with the matched tumor specimens for both the hypermutated and non-hypermutated patients (median = 0.89 frequency of concordance ranging from 0.77 to 0.94). **S3B. See also Table S3D.**Total mutational load and mutational signatures of derivatives (*n* = 71) and paired primary tumors (*n* = 11). The different colors represent 30 different signatures. **S3C.** Phylogenetic trees illustrating the evolutionary trajectory of SNU-4146 patient including both SNU-4146S4 and SNU-4146S4T. Based on the multiregional WES profiles, Treeomics classified somatic mutations as trunk (orange), shared (blue), and individual (green). Numbers on top of each branch indicate the number of acquired variants. Genes that are listed in the CGC are shown in orange. Numbers on the bottom of each branch indicate the estimated support values. The Treeomics statistical settings were as follows (sequencing error rate = 0.005, prior absent probability = 0.5, max absent VAF = 0.05, LOH frequency = 0, false discovery rate = 0.05, false-positive rate = 0.005, and absent classification minimum coverage = 100).

Figure S4

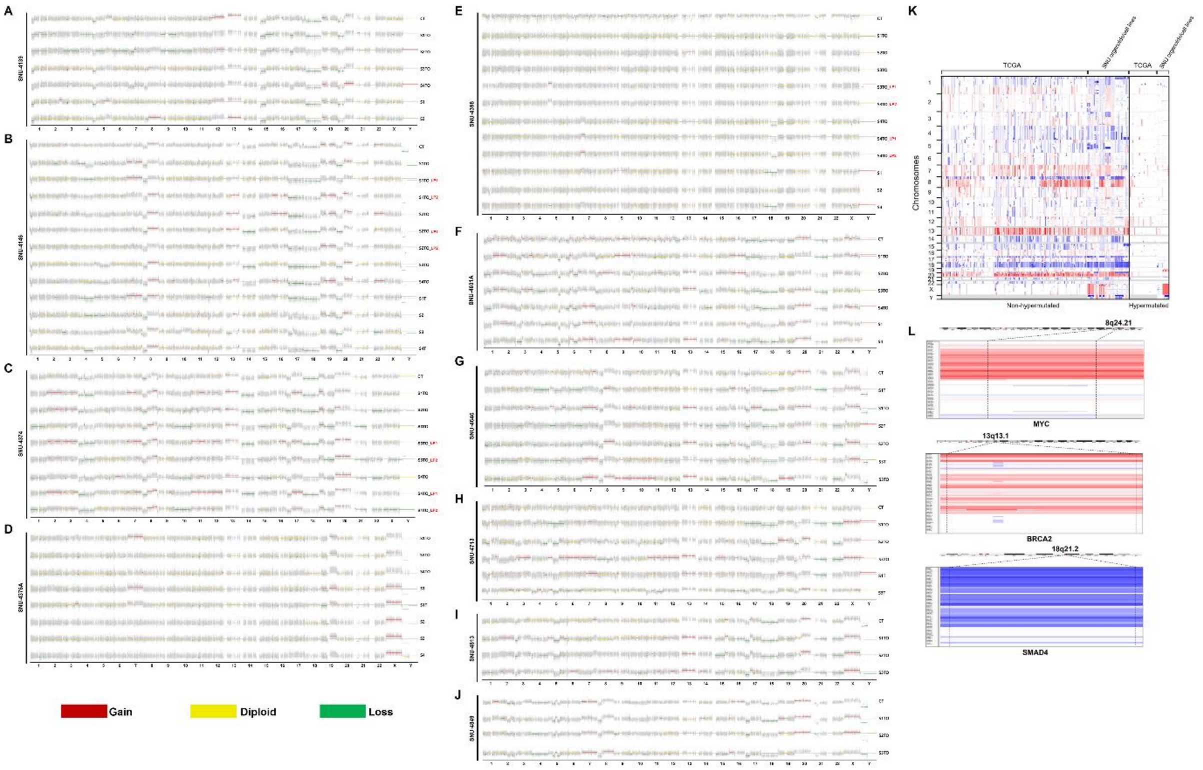

Figure S4A-J. Genome-wide gene copy number variations (CNVs) of CRC derivatives and paired primary tumors (red, gains; green, losses; yellow, diploid). **S4K.** Comparison of somatic copy number alterations found in our samples with TCGA COAD cohorts for both hypermutated and non-hypermutated cases. (red, gains; blue, losses; white, diploid). **S4L.** Somatic copy number alterations in organoids among frequently amplified genes identified in TCGA COAD. Manual inspection of the top regions revealed the presence of MYC- and BRCA2-amplified and SMAD4-depleted organoids, as well as a reported gain of 13q in the non-hypermutated group.

Figure S5

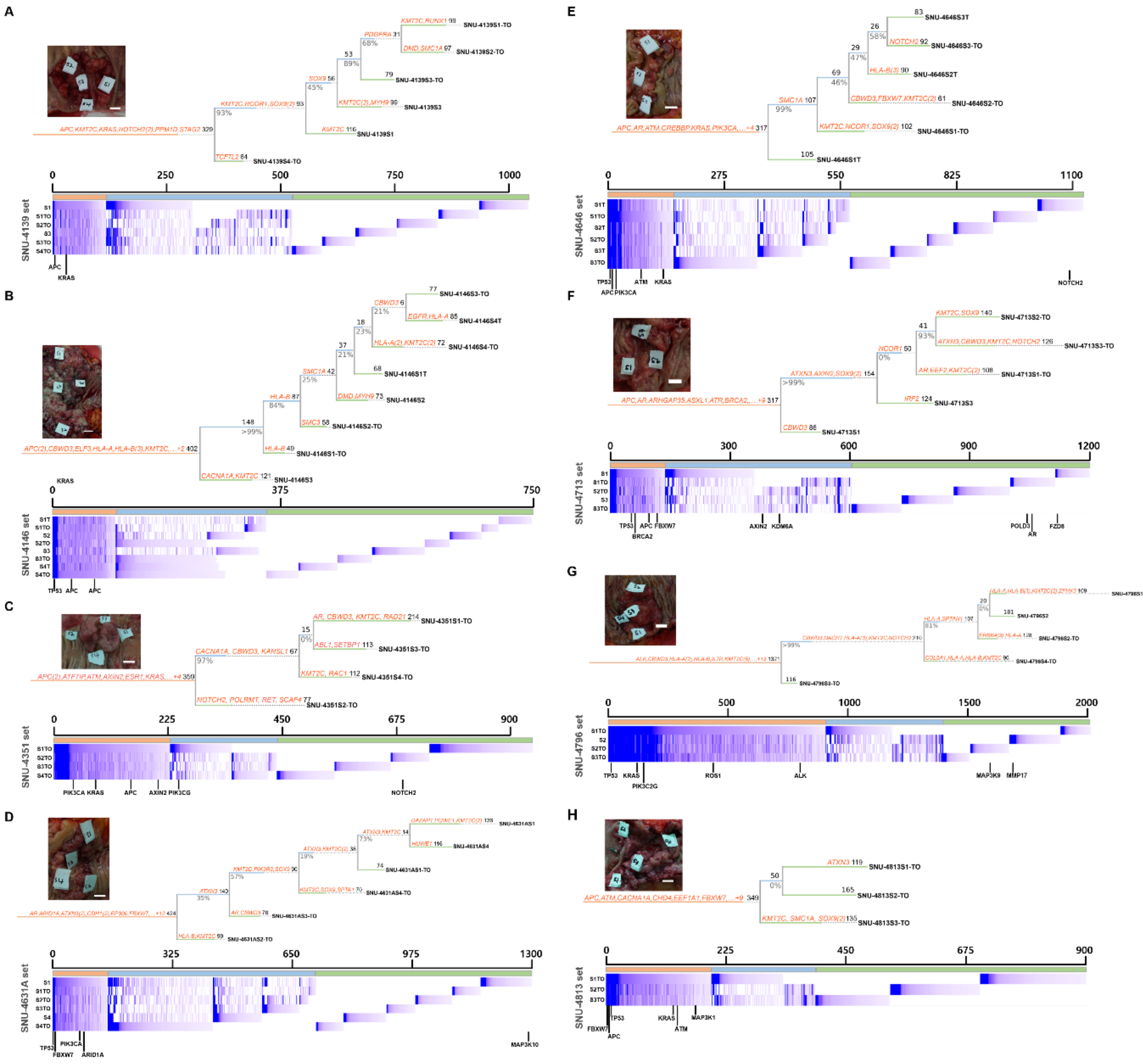

Figure S5A-H. Phylogenetic trees illustrating the evolutionary history of CRC-derived sets. Based on multiregional WES profiles, Treeomics classified somatic mutations as all (trunk), more than two samples (shared), and single samples (individual). Numbers on top of each branch indicate the number of acquired variants (including likely driver gene mutations, marked in orange). Numbers on the bottom of each branch indicate the estimated support values. The actual photograph of each tumor shows the marked sites from which each subclonal derivative was obtained (S1 – S4). Scale bars = 1 cm. Multiregional variant allele frequencies (VAFs) are shown as a heatmap. The three different classes of mutations (trunk,

shared, and individual) are indicated by the colored bars on the top. Driver mutations are indicated below each heatmap. The Treeomics statistical settings were as follows (sequencing error rate = 0.005, prior absent probability = 0.5, max absent VAF = 0.05, LOH frequency = 0, false discovery rate = 0.05, false-positive rate = 0.005, and absent classification minimum coverage = 100).

Figure S6

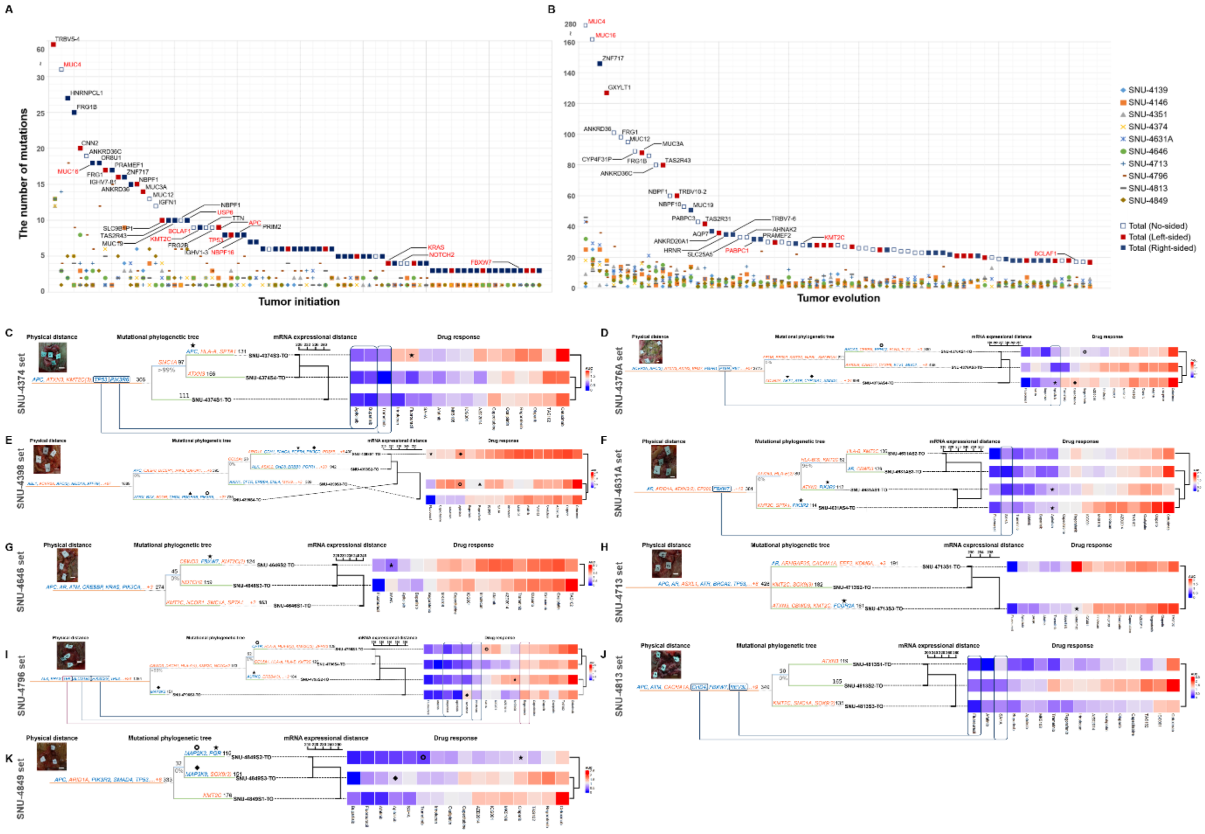

Figure S6A. See also Table S4A. Genetic mutations accounted for tumor initiation (*n* = 78). The tumor initiating genes were also sub-classified with the left-sided (*n* = 18), right-sided (*n* = 48) and no-sided (*n* = 12) mutations. **S6B. See also Table S4B.** Genetic mutations accounted for tumor evolution (*n* = 444)**.** The genes accounting for tumor evolution were also sub-classified with the left-sided (*n* = 156), right-sided (*n* = 214) and no-sided (*n* = 74) mutations. Genes present in the Vogelgram, such as *APC*, *TP53*, *KRAS*, and *FBXW7*, were exclusively identified among the tumor-initiating genes, which indicated that Vogelgram mutations mainly drive the initiation of tumors and had less influence on the subclonal evolution in our samples. **S6C-K.** Subclonal PDOs revealed heterogeneous drug responses caused by mutational ITH. Drugs that have analogous responses to all subclones such as bevacizumab and phytochemicals were excluded from the analysis. Genes that are listed in

the Cancer Gene Census (CGC) are shown in red, and genes that are associated with sensitivities of certain drugs are shown in blue on the mutational phylogenetic tree. Mutations observed in the trunk were associated with uniform responses among the subclones (marked with red lines, poor response; blue lines, good response, 14 trunk genes observed in 8 sets out of 12). Subclonal genetic mutations were associated to heterogenous drug responses (marked with plane figures, 30 cases observed in 11 sets out of 12). The Treeomics statistical settings were as follows (sequencing error rate = 0.005, prior absent probability = 0.5, max absent VAF = 0.05, LOH frequency = 0, false discovery rate = 0.05, false-positive rate = 0.005, and absent classification minimum coverage = 100).

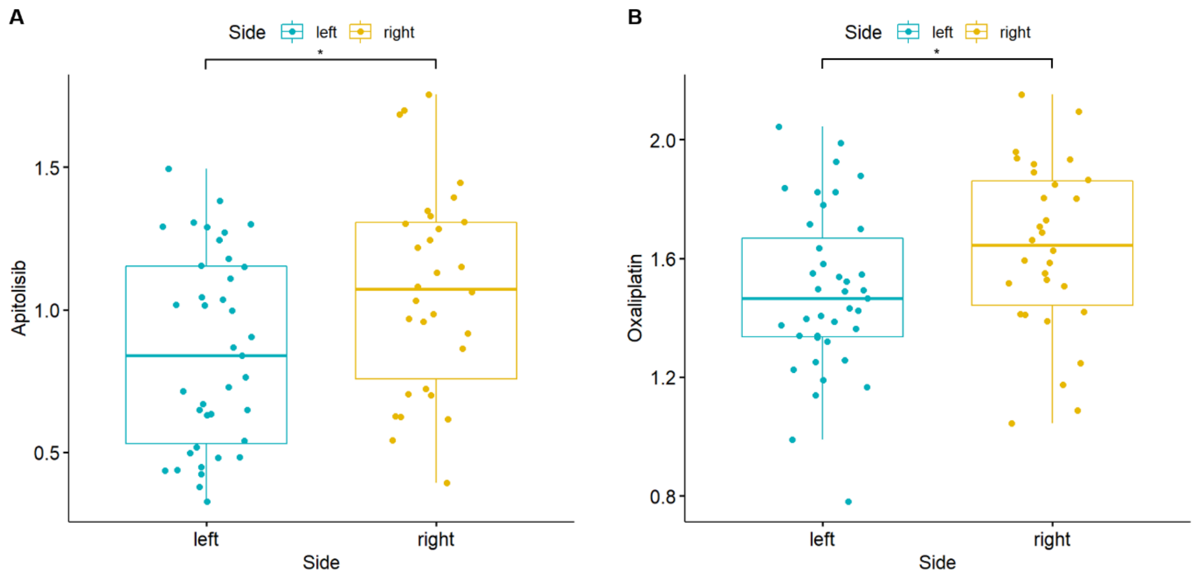

Figure S7A. See also Table S6C. Box plot of the AUC values of apitolisib categorized with left-sided (n = 39) and right-sided (n = 30) tumors. Apitolisib exhibited better responses in left-sided tumor in our samples. **S7B. See also Table S6C.** Box plot of the AUC values of oxaliplatin divided into left-sided (n = 39) and right-sided (n = 30) tumors. Oxaliplatin exhibited better responses in left-sided tumor in our samples. A value of $p < 0.05$ was considered statistically significant. * $p < 0.05$.
